# Using preregistration as a tool for transparent fNIRS study design

**DOI:** 10.1117/1.NPh.10.2.023515

**Published:** 2023-03-08

**Authors:** Philipp A. Schroeder, Christina Artemenko, Jessica E. Kosie, Helena Cockx, Katharina Stute, João Pereira, Franziska Klein, David M. A. Mehler

**Affiliations:** a University of Tuebingen, Department of Psychology, Faculty of Science, Tuebingen, Germany; b Princeton University, Social and Natural Sciences, Department of Psychology, Princeton, New Jersey, United States; c Radboud University, Donders Institute for Brain, Cognition and Behaviour, Biophysics Department, Faculty of Science, Nijmegen, The Netherlands; d Chemnitz University of Technology, Institute of Human Movement Science and Health, Faculty of Behavioural and Social Sciences, Chemnitz, Germany; e University of Coimbra, Coimbra Institute for Biomedical Imaging and Translational Research, Coimbra, Portugal; f University of Oldenburg, Department of Psychology, Neurocognition and functional Neurorehabilitation Group, Oldenburg (Oldb), Germany; g RWTH Aachen University, Medical School, Department of Psychiatry, Psychotherapy and Psychosomatics, Aachen, Germany; h University of Münster, Institute for Translational Psychiatry, Medical School, Münster, Germany

**Keywords:** functional near-infrared spectroscopy, study design, preregistration, guide, template, open science

## Abstract

**Significance:**

The expansion of functional near-infrared spectroscopy (fNIRS) methodology and analysis tools gives rise to various design and analytical decisions that researchers have to make. Several recent efforts have developed guidelines for preprocessing, analyzing, and reporting practices. For the planning stage of fNIRS studies, similar guidance is desirable. Study preregistration helps researchers to transparently document study protocols before conducting the study, including materials, methods, and analyses, and thus, others to verify, understand, and reproduce a study. Preregistration can thus serve as a useful tool for transparent, careful, and comprehensive fNIRS study design.

**Aim:**

We aim to create a guide on the design and analysis steps involved in fNIRS studies and to provide a preregistration template specified for fNIRS studies.

**Approach:**

The presented preregistration guide has a strong focus on fNIRS specific requirements, and the associated template provides examples based on continuous-wave (CW) fNIRS studies conducted in humans. These can, however, be extended to other types of fNIRS studies.

**Results:**

On a step-by-step basis, we walk the fNIRS user through key methodological and analysis-related aspects central to a comprehensive fNIRS study design. These include items specific to the design of CW, task-based fNIRS studies, but also sections that are of general importance, including an in-depth elaboration on sample size planning.

**Conclusions:**

Our guide introduces these open science tools to the fNIRS community, providing researchers with an overview of key design aspects and specification recommendations for comprehensive study planning. As such it can be used as a template to preregister fNIRS studies or merely as a tool for transparent fNIRS study design.

## 1 Introduction

The design and analysis of hemodynamic brain-imaging studies encompasses many degrees of freedom in terms of parameter choices for data acquisition, preprocessing, and analysis.^1^^,^^2^ Replicability and reproducibility issues due to undisclosed analytical flexibility have been intensively discussed and demonstrated for several brain imaging techniques, including hemodynamic functional magnetic resonance imaging (fMRI)^3^^–^^5^ and electroencephalography.^6^^,^^7^ Similarly, the impact of different design and analytical choices in functional near-infrared spectroscopy (fNIRS) research has recently been a focus.^8^^,^^9^ When undisclosed, these bear the risk of questionable research practices (QRPs) and may imperil the reproducibility and replicability of published results.^10^ Addressing these widely pervasive concerns requires adopting transparent study designs, reporting, and other highly encouraged open-research practices that researchers across fields have developed. These include sharing materials, data, and code, releasing openly accessible preprints, and publishing a preregistration protocol.^11^^–^^14^^,^^15^ In particular, study preregistration has been recently highlighted as an important best practice tool for functional neuroimaging,^3^^–^^6^^,^^16^^,^^17^ including fNIRS research.^18^^–^^20^ However, drafting a preregistration protocol can feel especially daunting for beginners as it requires formulating a detailed *a priori* study and analysis plan,^14^^,^^21^ especially in a field with frequent methodological and technical advances that make any attempt to standardize procedures challenging.

**Fig. 1:**
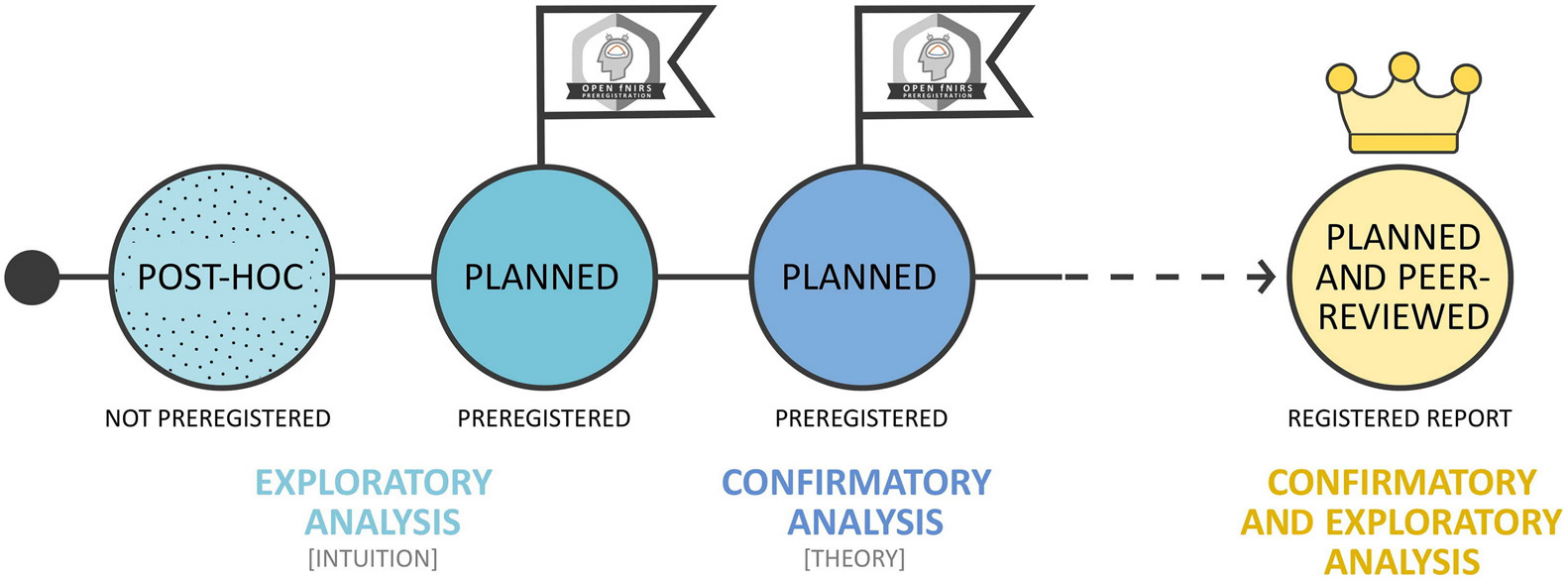
Overview of continuum between mostly intuition based (i.e., rather exploratory) and mostly theory based (i.e., rather confirmatory) analyses and their relation to study preregistration and Registered Reports.^23^

Preregistration protocols are time-stamped documents in which researchers specify their hypotheses and plans for data collection, preprocessing, and analysis typically before data collection starts.^13^^,^^22^ This procedure allows for transparency regarding which aspects of the study were decided before (i.e., planned) or after (i.e., post-hoc) data collection and, thus, evaluating where the study falls on the spectrum between confirmatory and exploratory research (Fig. 1). Preregistration is expected to effectively prevent undisclosed analytical flexibility, including (unintentional) p-hacking, cherry-picking of results, and certain forms of hypothesizing after the results are known (HARKing).^10^^,^^24^^–^^28^ Preregistrations are made publicly available before data collection or can be embargoed for a specific period of time on websites such as the Open Science Framework (OSF; osf.io). An even more compelling variant of preregistration is a Registered Report, which in its essence is a peer-reviewed preregistration with in-principle acceptance. This publishing format is increasingly offered at peer-reviewed journals^29^ (or independent of journals, e.g., Peer Community In Registered Reports^30^) and involves an initial stage 1 peer review based on the proposed methodology, hypotheses, analysis, and sampling plan. Successful submissions are accepted-in-principle before the research is conducted and irrespective of the study’s outcome. Registered Reports are published following a final stage 2 peer review to assess whether the research was conducted according to the preregistered research protocol. Thereby, the format aims to mitigate researcher and publication biases.

The first meta-analyses of preregistered research showed that reported effect size estimates are indeed substantially smaller,^31^^,^^32^ while the proportion of reported non-significant results^14^^,^^33^ is substantially larger than those reported for non-preregistered research. Taken together, these findings may indicate that the process mitigates publication bias and other QRPs that inflate effect sizes and result in false positive findings. Thus, it has been advocated that preregistration benefits both the scientist directly and the field more broadly.^13^^,^^14^^,^^22^^,^^27^^,^^34^ For example, preregistration can be a planning process to help researchers and their collaborators think through decisions in advance in a structured way as the process involves documenting hypotheses before the data were seen (i.e., *a priori*) and designing analysis and sampling plans accordingly. It can thereby protect the researcher from implicit biases that may arise during data analysis (e.g., incorrectly recalling that the only significant effect is the one that was originally predicted). In fNIRS research, several recent efforts have been made to develop guidelines for preprocessing and analyzing fNIRS data,^9^^,^^35^^,^^36^ as well as for reporting practices.^19^ Similar guidance would be desirable for the planning stage of an fNIRS experiment. For the field, preregistration is a transparency tool that allows others to verify, understand, and reproduce a study. It can thereby increase the perceived quality of research and may also enhance the trust that researchers have in both their own and others’ research.^14^^,^^21^^,^^27^^,^^37^^,^^38^

To make the task of writing a preregistration protocol more tractable, proponents of open science have developed guides, checklists, and templates to facilitate transparent study design, preregistration, and reporting for various types of research including neuroimaging.^7^^,^^39^^–^^45^ For fNIRS research, however, no similar resources exist yet, and they are highly desired.^18^

In what follows, we share and discuss a comprehensive preregistration guide and template for continuous-wave (CW), task-based fNIRS experiments that were developed following the 2021 hybrid fNIRS summer school hosted by the University of Tübingen.^46^ Specifically, this preregistration guide walks the fNIRS user through specific design and analysis considerations. Further, we adapted and extended the previously established psychological research preregistration-quantitative template^34^ toward fNIRS research-specific needs, which are elaborated in the respective sections. For a better illustration, we provide examples and link the corresponding text sections below to the items of the adapted preregistration template. We note that the examples may also include technical details, which should not be considered to be recommendations regarding fNIRS methodology itself but are only included to exemplify the desired level of detail for respective preregistration items. The preregistration template file can be accessed via an OSF project page,^55^ and readers are welcome to contribute for future extensions.

**Fig. 2:**
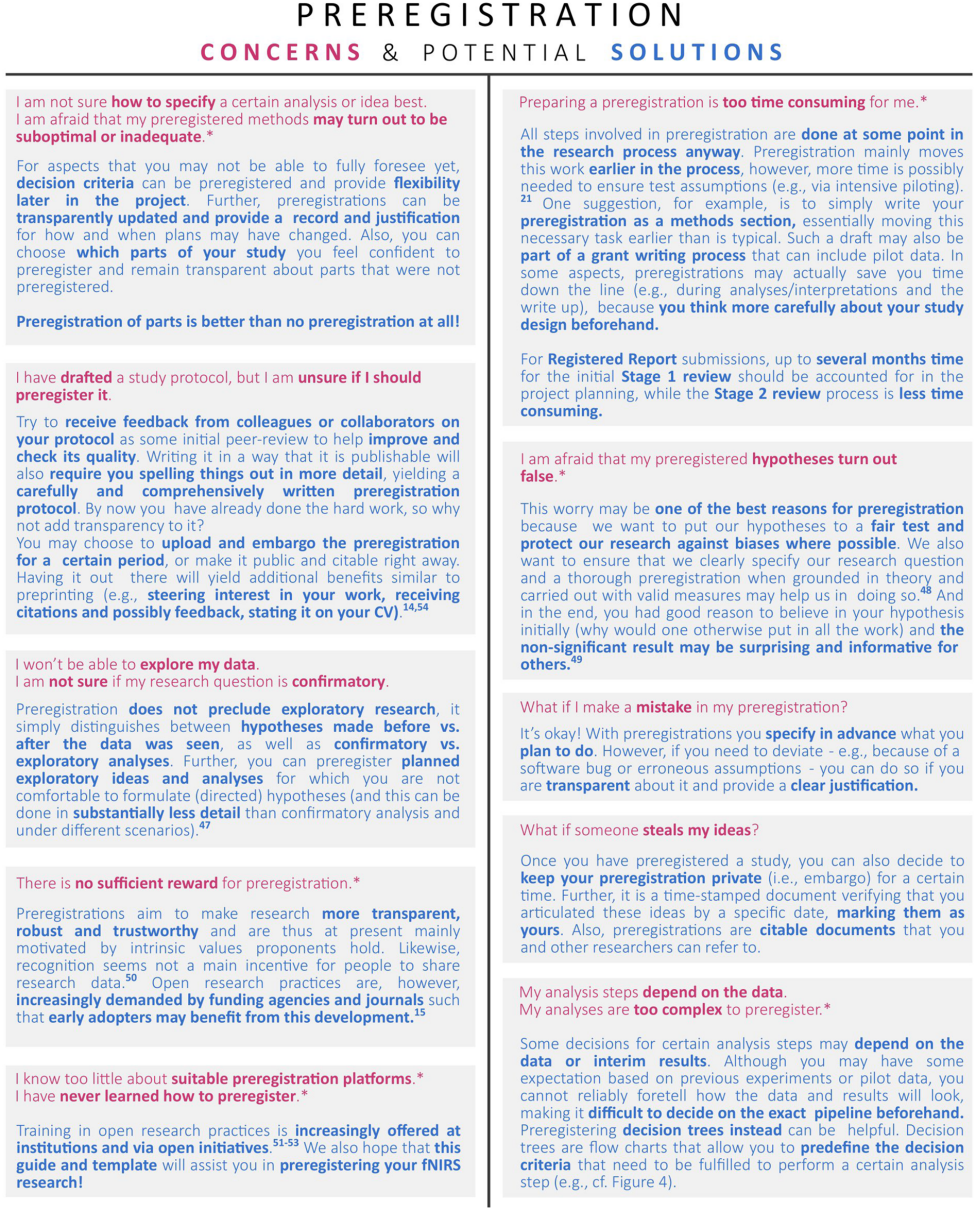
Common preregistration concerns and potential solutions.^14^^,^^15^^,^^47^^–^^54^ Those indexed by “*” have recently been identified in a survey conducted with researchers working in the field of functional neuroimaging.^21^

We further wish to highlight that the overall goal of preregistration is to maximize transparency as much as possible, not to write the perfect and most complete preregistration. For some researchers, especially those who write their first preregistration or who conduct their first fNIRS study, this may mean that they may only be able to fill in details for those sections of the preregistration template that are most addressable for the current study, while stating explicitly for which sections sufficient knowledge about study details at the point of conceptualization is still lacking. Others may choose not to submit a preregistration at all, but instead to use this guide and the template as a tool for designing an fNIRS study. Although we encourage researchers to publish preregistrations of their fNIRS studies whenever possible, we have attempted to make this template useful for those who wish to do either. For researchers who are still hesitant to preregister, we discuss some common concerns and suggestions for potential solutions in Fig. 2. Further, we note that, although this guide and the associated template were created with the aim to assist researchers in designing their fNIRS studies, they can also support evaluating these (e.g., as part of a literature search, systematic review, or manuscript submission review process).

Finally, we note that with recent optical engineering and methodological advances, there is an increasing demand for new analytic methodologies and procedures.^56^ This bears one particular preregistration concern relevant for fNIRS: with regards to preprocessing and data analysis, it may be possible that approaches considered state-of-the-art at the time of preregistration may seem somewhat outdated at a later study phase. This possibility can be daunting for fNIRS researchers generally and for beginners in the field in particular. However, it can be mitigated when researchers disclose and justify methodological deviations from the preregistered protocol in the final manuscript or report updated analyses in addition to the preregistered analyses.

## 2 Step-by-Step fNIRS Study Design

**Fig. 3:**
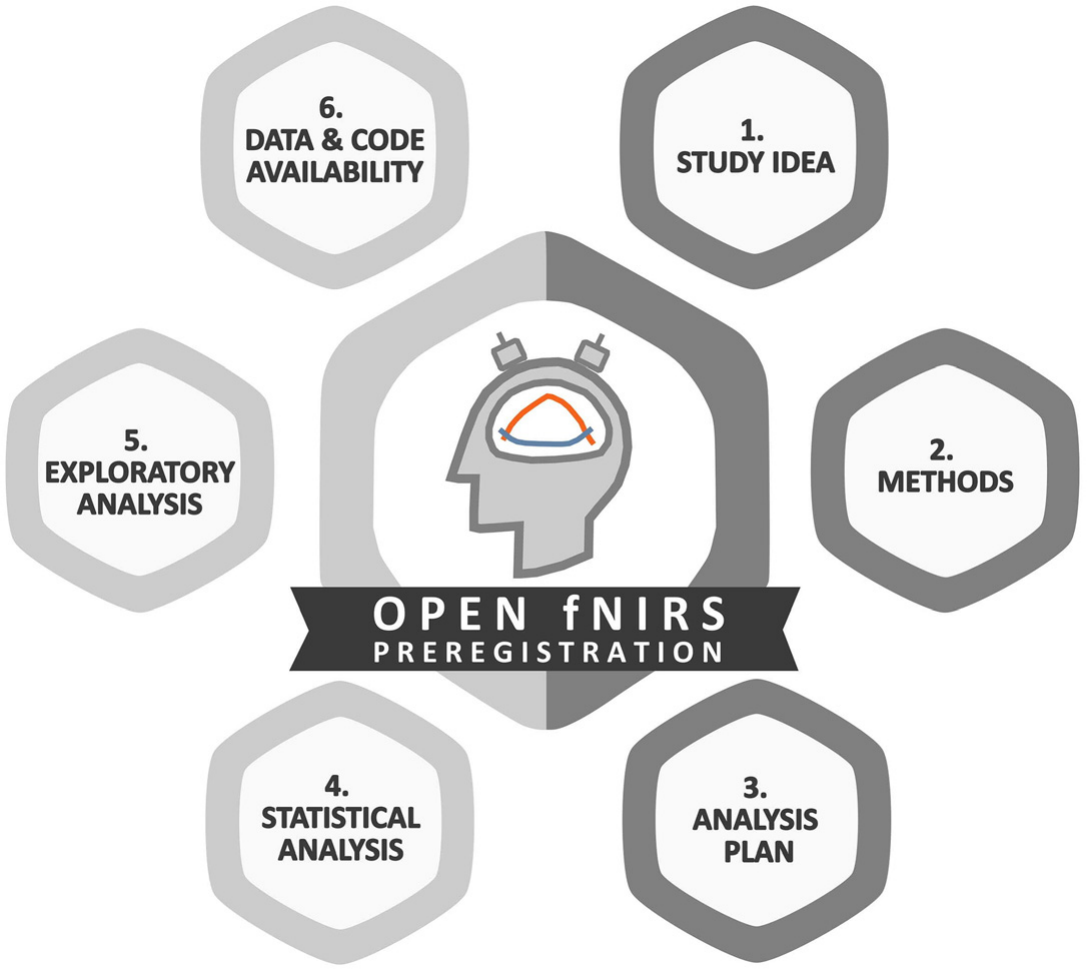
Overview of the fNIRS study design guide structure.

In the following, we provide a comprehensive step-by-step preregistration guide for transparent fNIRS study design as shown in Fig. 3. The guide covers all steps from generating a study idea (cf., Sec. 2.1) to publishing research outcomes, including open access to data and code (cf., Sec. 2.6), the corresponding items of the complementary preregistration template are referenced. It aims to aid researchers getting started with preregistering an fNIRS study.

### 2.1 Study Idea

The idea of a study comes first. To conceptualize a new study, researchers conventionally start with reviewing what is already known, i.e., they build up background knowledge (based on existing literature). This process is ideally guided by genuine interest and allows researchers to identify knowledge gaps. To fill in such a gap requires a specific study motivation (“Why is it important to address this problem?”) and a justification (“Why is fNIRS an appropriate method for this study?”). Researchers should consider the possible scope of their study: ideally, the study should tell a specific story; otherwise, if the scope goes beyond a single story, it will likely require defining a set of experiments or even a whole research program on this topic. Importantly, in addition to original empirical and replication studies, researchers can also preregister methodological studies (e.g., comparison of different motion artifact correction algorithms), exploratory studies,^47^ or secondary analyses of existing data sets.^40^^,^^57^

The motivation and justification for a study provide its rationale. They lay the groundwork for formulating research questions and generating hypotheses based on theory and previous findings. Thereby, preregistration allows for evaluation of whether a research question and analysis is rather confirmatory (i.e., mostly theory-based) or rather exploratory (i.e., mostly intuition-based) as shown in Fig. 1.^26^ In confirmatory research, hypotheses are defined in advance to test a specific relation between the variables (e.g., activation in area A is larger in condition C1 than C2). Confirmatory hypotheses are the basis of designing a sampling and analysis plan (whereas post-hoc exploratory analyses can follow confirmatory analyses). In exploratory research, open questions or hypotheses with alternative outcomes under different assumptions can be stated.^47^ It is worth noting, however, that exploratory analyses can also be planned and preregistered.

Preregistration templates vary in the extent to which the study idea, theoretical background, and previous results need to be articulated. We recommend providing sufficient theoretical information to comprehend the study’s goals and the adequacy of investigating the research question with fNIRS.

Preregistration template item(s): T1, T9, I1/A1, I2/A2, I3, and I4.

### 2.2 Methods

#### 2.2.1 Study design

The study design includes within-subject factors (e.g., different conditions in a cognitive or motor task) and, where applicable, between-subject factors (e.g., different groups such as patients and healthy controls or different training groups). fNIRS will be measured during a certain task (e.g., task-related activation or intervention-induced activation changes) or during rest (e.g., resting-state activation or functional connectivity). For a given task, the effect of interest should be operationalized as the difference between conditions of interest (see Box 2 in Ref. 58). Additionally, control variables (e.g., tests and questionnaires) and localizer tasks can be considered.

Task-based conditions are usually presented in a block design, an event-related design, or a mixed block/event-related design. The number of blocks/trials, the duration between the blocks/trials, and the order of trials/blocks determine the signal-to-noise ratio (SNR). These parameters can be explicitly stated in a preregistration, for example: “In this study, a block design of 10 blocks per condition was presented in a fixed order with a duration of 15 s and a corresponding jittered inter-block-interval of 20 to 23 s.” or “In this study, an event-related design was realized with 30 trials per condition in a randomized order with a duration of 2 s and a jittered inter-trial-interval of 10 to 15 s.” or “In this study, a rapid event-related design was realized with 15 trials per condition presented in a randomized order with a duration of 5 s and a jittered inter-trial-interval of 2 to 3.5 s.”

Preregistration template item(s): A4, M11, M12, M14, and M15

#### 2.2.2 Sample characteristics

A preregistration specifies the characteristics of the sample. This includes brief information about the type of sample that will be recruited (e.g., healthy population, specific clinical population, specific age group, etc.). These details are relevant as they may impact any behavioral but also fNIRS effects, e.g., due to atrophic processes or changes in hemodynamics that are related to aging or a pathological condition. If the sample is divided into groups, objective criteria or randomization procedures should be defined. Possible confounders inherent to groups and how to address these (e.g., motion artifacts in children versus adolescents or patients versus healthy controls) should be considered. For instance, sample characteristics such as age or clinical status may impact fNIRS data analysis plans.^19^^,^^59^^,^^60^

An fNIRS preregistration should further specify study-specific inclusion and exclusion criteria in accordance with the study’s ethical review board. Basic demographic information should be collected and reported, if possible (e.g., age, gender, ethnicity, head size, hair color, absence of hair, and skin pigmentation). Although including health information is likely informative and encouraged, sharing such information should be done with care to prevent harming the privacy and data protection of participants, who must remain anonymous. In cases in which sharing sensitive data is not required or adequate, such personal data may still be relevant for screening exclusion criteria. If sample characteristics are relevant for the purpose of the study, this should be apparent in the hypotheses.

NIR light transmission is affected by optical properties such as skin pigmentation, hair thickness, and cortical thickness. The impact and correction of racial bias in fNIRS and other oxygen-based measurements is an open topic that still needs further evaluation.^61^^–^^64^ Researchers should be aware of this discussion and weigh the possible benefits of inclusive study designs (e.g., better generalizability, the possibility to include moderators in a large sample) against less inclusive designs (e.g., the possibility of a better signal quality) along with ethical considerations.

Preregistration template item(s): AP1-AP3 and M12

#### 2.2.3 Sample size planning

The sample size (i.e., the final number of individuals included in a study) should be determined in the study design phase as it is crucial for answering the research question. Justifying the choice of sample sizes is essential in an adequate study design as it provides transparency about the criterion used to determine when data collection is completed.^65^^,^^66^ Ideally, justifications should account for potential drop-out and data censoring (e.g., due to compromised/missing data or behavioral performance). Data drop-out/censoring may be considerable for fNIRS research, depending on the context (e.g., for clinical or infant studies).

Transparent sample size justifications are pivotal to allow for evaluating the precision and hence robustness of effects that a study is trying to detect. Different justification procedures exist: conducting an *a priori* power or sensitivity analysis is conventionally recommended as best practice for confirmatory research when employing inferential statistics.^3^ In reality, however, sampling plans may often be determined by external factors such as resource constraints (e.g., time, funding or sample characteristics), which reflect another, albeit limited, justification for sample size.^65^ Often resource constraints and heuristics result in too small sample sizes, leading to lower statistical power and thus over or underestimation of true effects due to imprecision in the outcome estimate. This is why small studies, in particular, are more likely to yield outcome estimates that are not as reliable and should thus not be a basis for future sample size calculations.^67^ Further, in the presence of publication bias, they can contribute to inflated effect size estimates in the literature.^68^

Putting *a priori* hypotheses to a fair confirmatory test requires sample size estimations that are informed by an adequate statistical power analysis based on realistic or relevant effect size assumptions.^29^ Statistical power describes the probability that a certain effect (size) can be detected, assuming that it exists. All relevant details of sample size estimation should be reported (i.e., assumed or targeted effect size, statistical model, assumptions on correlational designs, and inferential parameters) in sufficient detail such that a power analysis can be reproduced. Alternatively, Bayesian sequential sampling plans allow for flexible stopping once a predefined evidence threshold in the form of a parameter estimate, or Bayes factor (following a Bayes factor design analysis), is reached (see for example Ref. 67). They should be informed by a Bayesian sensitivity analysis, which also relies on specifying an expected effect under the alternative (and null) hypothesis. Further, they take into account the uncertainty around this point estimate via the use of a prior distribution that covers a range of plausible effect sizes.^69^^,^^70^ Although Bayesian approaches are far less used, they offer the main advantages that they are more resource efficient and allow for sampling until sufficient evidence for the absence of an effect is accumulated.^69^^,^^70^ Bayesian sampling plans should be reported in sufficient detail, including the assumed effect size, prior distribution, and stopping criterion in the form of a posterior interval or Bayes factor for the alternative and null hypotheses.^71^^,^^72^

Frequentist and Bayesian sample size estimations both require specifying a minimum effect size that the used statistical test aims to detect with a specific power or sensitivity, respectively. Several open-source tools are available for power analysis computations (see Table 1). Researchers should provide a rationale for the effect size estimate that is included in their power or sensitivity analysis. Desired power or sensitivity thresholds are commonly set to 80% or 90%, depending on the field and context. Importantly, the relationship between the targeted effect size and its corresponding sample size and statistical power is curve shaped.^86^ Thus, for instance, even small deviations between targeted versus observed effect sizes may result in a substantial loss of statistical power. Traditionally, effect size estimates are drawn from previous, comparable research. For example, if a previous published fNIRS study reported a medium effect size of f=0.25 for interpersonal brain synchronization,^87^ with an acceptable power of 1− b=0.8 and a significance level of 0.05, replication of the effect in a paired samples t-test of HbO mean amplitude in the predefined region of interest (ROI) would require n=34 participants. For more complicated tests that might include interaction terms with other factors such as hemisphere, channels, other ROIs, or conditions, it can be expedient to model the expected pattern of results^73^; see M3 for an example with annotated R code. However, we advise caution with this approach given the concerns described in the next paragraph. We further note that, in principle, results from fMRI could also inform about the range of an anticipated effect size. However, the sensitivity of fNIRS to deeper cortical regions is usually weaker and depends on the probe as well as on other system and design characteristics such as the SNR and experimental design. We are not aware of current standards for power analysis in fNIRS research and warrant that effect sizes will be different for specific populations (adults, children, patient populations), for different setups (e.g., fNIRS versus diffuse optical tomography), and parameters implied in experimental design (e.g., different inter-stimulus intervals in event-related design^88^). If an effect size estimate is available, researchers should specify the dependent variable [e.g., measure in the ROI/channel(s)], clearly report the statistical model and effect of interest for the sample size justification (e.g., the main effect in a 2×2 mixed design) and the implicated type I and type II error rates, as well as possible correction methods.

**Table 1:** Open-source tools to conduct power and sensitivity analyses.

Name	Description	Link
InteractionPoweR	R library for power analysis of interaction effects.	https://github.com/dbaranger/InteractionPoweR
ANOVA power shiny app^73^	Monte Carlo simulations of factorial experimental designs to estimate power for an ANOVA and follow-up pairwise comparisons.	Ref. 74
Brainpower	List with tools for power analyses, in particular for fMRI studies.	Ref. 75
Bayes factor design analysis^69^^,^^70^	R library to conduct Bayesian sensitivity analyses and sampling plans.	https://github.com/nicebread/BFDA
Superpower	E-book, R library, and Shiny apps for power analysis in factorial experimental designs.	Ref. 76
Jamovi^77^	Open-source software to conduct informative power and sensitivity analyses for t-test family (jpower plugin), including power curve illustrations.^68^	Ref. 78
G*power^79^^,^^80^	Widely used open-source software to conduct power and sensitivity analyses for commonly used statistical tests and more advanced statistical models.	Ref. 81
pwr	R library for commonly used statistical tests.	Ref. 82
More Power	GUI for commonly used statistical tests and more advanced statistical models.	Ref. 83
SampleSizePlanner^84^	R library for different sample size planning strategies and shiny app	Ref. 85

Further, researchers should take into account that effect sizes reported in non-preregistered studies and meta-analyses—in particular those that report significant findings—are more likely to be affected by publication bias, small sample sizes, and researcher bias, and they thus more likely overestimate true effect sizes. For instance, recent meta-research found overestimations of a factor of two to three when compared with preregistered research.^31^^,^^32^^,^^68^ It is thus recommended to either adjust for potential inflation in reported effect size estimates^89^ or refer exclusively to literature that is unlikely to be affected by publication bias and is sufficiently powered (e.g., meta-analyses based on Registered Reports and sufficiently large samples with high precision in reported effect size estimates).

Alternatively, an effect size of interest without prior empirical results could be also the smallest effect size of interest (SESOI), which expresses the smallest relevant effect that the researchers care about (e.g., clinical significance and theoretical implication^65^). Researchers should justify their choice of SESOI and may use anchors specific to their research question. For instance, when powering for group or condition differences, other measures (e.g., clinical or behavioral) can be used as an anchor for a SESOI. To provide an example from clinical research, for instance, it has been estimated that improvements in depressive symptoms may need to exceed a standardized mean difference (SMD) of at least 0.24 to be considered a clinically meaningful change. If the goal of fNIRS recordings in the context of a depression trial is to detect accompanying hemodynamic correlates as a potential biomarker that can indicate clinically relevant improvements of depressive symptoms, the SESOI for hypothesized hemodynamic changes assessed by fNIRS should be at least SMD=0.24^90^ to be sufficiently sensitive and thus informative. An alternative anchor for SESOIs of hemodynamic effects may be effect sizes that are observed for behavioral effects. For instance, rates of anticipated non-responders (e.g., percentages of successful trials) can inform the lower effect size boundary. Using sampling plans that are based on SESOIs, it is more likely to detect meaningful effects. Moreover, in the case of non-significant/inconclusive results, they are more likely to yield evidence for the absence of an effect through follow-up equivalence tests (or their Bayesian equivalent) that allow for rejecting the alternative hypothesis.^48^^–^^92^ In the context of hemodynamic brain imaging, such equivalence tests have been recently discussed for fMRI applications.^93^

Sample size planning of any form described above should be done at least for the primary outcome measures used for the critical hypothesis test of a study. In case other tests are also planned (e.g., secondary outcomes and manipulation checks), researchers are free to calculate and report multiple sample size estimations and determine the most conservative sample size estimate.

To assist researchers in performing adequate sample size planning, we provide an overview of open-source software tools (cf., Table 1).

Preregistration template item(s): M3 and AP8

#### 2.2.4 Instrumentation

In general, a detailed description of the fNIRS device including the name of the system and the manufacturer should be provided. Further, the specific fNIRS optode placement settings are determined by the used fNIRS device and to a lesser extent by several researcher decisions. For instance, different options for attaching the optodes to a head cap (e.g., within an fNIRS cap or a headband, self-printed) exist and should be reported. Moreover, the type and number of sources (i.e., LED versus laser), detectors (i.e., silicon photodiode versus avalanche photodiode), and short-distance detectors, including the corresponding source-detector distances (e.g., ∼3  cm for long-separation channels and ∼0.8  cm for short-separation channels for adults^94^), should be documented. If planned, auxiliary measurements integrated into the head cap (e.g., accelerometer) and peripheral physiological measurements (e.g., blood pressure, electrocardiogram, and plethysmography) should be documented together with a motivation for their recording [e.g., as nuisance regressors in the general linear model (GLM)]. If any additional measurements [e.g., electroencephalography (EEG), eye-tracking, and transcranial electric stimulation] will be taken during the experiment, the preregistration should include how the data across different measurement devices will be synchronized (e.g., via lab-streaming layer).

Preregistration template item(s): M9 and M10

#### 2.2.5 Optode array design and optode placement

fNIRS recordings are limited to superficial cortical layers with a spatial resolution of around 2 to 3 cm and a penetration depth of around 1.5 to 2 cm into the cerebral cortex.^36^^,^^56^^,^^95^ Cortical brain activity is measured by fNIRS from the brain area over which the optodes (i.e., sources and detectors) are placed. Of note, the distance between optodes and underlying brain regions may be variable, depending on the brain region that is targeted, and the amount of extra-cerebral tissue present between the optode and the respective cortical area. In addition, optode placement at a certain ROI can be challenging due to lacking anatomical information. Accordingly, designing an optode array (i.e., optode arrangement) in relation to anatomical landmarks and/or standardized locations (e.g., 10 to 20 EEG locations^96^^,^^97^) is essential for every fNIRS study to enhance reproducibility and replicability. It is noteworthy, however, that the position of channels relative to the scalp can also be variable between setups or even recording sessions.^98^^,^^99^ From a practical point of view, any arrangements for reducing potential discomfort of the participant caused by scalp-optode contact (e.g., different spring holder pressures) and additional materials used during participant preparation (e.g., use of a cotton swab to move the hair away, use of a light-shielding overcap) should be reported.

The most accurate optode array design results from individual anatomical information in combination with individual 3D optode coordinates that are obtained by digitization, photogrammetry-based,^100^^–^^102^ and neuronavigation tools.^103^^–^^105^ The precision of this procedure can be further improved by incorporating functional MRI data of the same participant or based on a probabilistic approach.^89^ If such procedures are anticipated, the applied tools or the performed custom-made steps for the co-registration should be reported. Alternatively, procedures that allow for the design of an optode array with respect to anatomical landmarks (e.g., nasion, inion, and left and right preauricular points) and to the standard EEG 10 to 20 positions^96^^,^^97^ are often applied. For this purpose, several software tools have been invented and validated by the fNIRS community [e.g., AtlasViewer,^106^ Array Designer,^107^ fNIRS optode location designer (fOLD),^108^ dev-fOLD,^109^ modular optode configuration analyzer (MOCA),^110^ and simple and timely optode registration method for functional near-infrared spectroscopy (STORM-Net)^111^].

In addition to the applied software, the used settings such as the brain parcellation atlas (e.g., AAL2^112^^,^^113^ and Brodmann^114^), the basis of anatomical landmarks (e.g., 10–20, 10–10, and 10–5 positions^96^^,^^97^), and the employed head model (e.g., Colin27^115^ and SPM12^116^) should be specified in the preregistration. Moreover, the software-specific input parameters and settings for the probe design should be documented in as much detail as necessary to be reproducible. For example, fOLD^108^ allows for settings such as brain atlas, the basis of anatomical landmarks, probe symmetry, and level of specificity (%) that are all necessary to reconstruct the optode array.

Preregistration template item(s): M10 and M13

### 2.3 Analysis Plan

#### 2.3.1 Data exclusion criteria

After data collection and before starting data analysis, the quality of the data needs to be ensured. To ensure sufficient fNIRS data quality, it is important to avoid the inclusion of channels with a poor signal quality. These often result from light instabilities due to poor optode-scalp coupling,^117^^,^^118^ which can influence the subsequent preprocessing steps. Therefore, the preregistration should define criteria for signal quality evaluation and data exclusion at the trial, channel (so-called pruning), and participant levels. The quality of the fNIRS signal, for example, can be evaluated by specific calculations (e.g., SNR, coefficient of variation,^119^ contrast-to-noise ratio, and scalp coupling index^19^^,^^120^) applied on a certain type of fNIRS data (raw density, optical density, or concentration data). Exclusion criteria should specify the critical measure (i.e., threshold value or qualitative measure), the type of exclusion (i.e., listwise versus casewise), and the level of exclusion (i.e., participant, channel, or trial). For example, studies might exclude channels with an SNR below 15 dB,^121^ participants with <50% usable channels,^122^ missing data or invalid cap placement,^123^ or trials based on incorrectly solved trials.^124^

Defining data exclusion criteria *a priori* may be challenging and is highly dependent on prior knowledge, making it particularly difficult for researchers new to fNIRS. This challenge can be overcome by performing initial pilot experiments that allow for the specification or change of values. Moreover, hemodynamic data exclusion criteria can be deduced from comparable fNIRS setups (when using identical fNIRS systems). Further, behavioral data exclusion criteria can be retrieved from basic studies with comparable tasks.

Preregistration template item(s): M7

#### 2.3.2 Data Preprocessing

An important step in fNIRS studies is the preprocessing of the data as one major issue of fNIRS measurements is the contamination with different noise components that are either related to motion (e.g., signal quality and motion artifacts^117^^,^^118^^,^^125^) or to systemic physiology (e.g., evoked and non-evoked cerebral and extracerebral systemic confounds.^36^^,^^126^ These artifacts constrain the main goal of task-related fNIRS research, namely, to restore the underlying hemodynamic response to a certain task.

Most of the preprocessing steps, are complex and different parameter selection and/or different orders of the applied preprocessing steps might lead to different results and thus to different interpretations of the data.^8^^,^^127^^,^^128^ For instance, some parameters might be device-specific (e.g., sampling rate, but also measurement units and scales) and should be interpreted relative to the technical setup. Furthermore, the implementation and parameters of similar preprocessing steps may vary slightly between different fNIRS analysis toolboxes; therefore, the used toolbox should be stated in preregistration protocols. We refer to other resources for an overview of existing fNIRS analysis toolboxes.^36^^,^^129^^–^^131^

As a result, researchers might adapt their preprocessing pipeline until they find the desired effect, which can increase type I error rates (i.e., the risk for false positive findings).^7^^,^^132^ To prevent results-based analytic decisions, the decisions regarding data preprocessing should be transparently documented *a priori* and ideally in a specific, precise, and exhaustive way.^7^^,^^24^ Accordingly, the preregistration of fNIRS data analysis steps should provide all details that are necessary to reproduce the whole pipeline without seeing any of the code (i.e., specific) so that each step is only interpretable in one direction (i.e., precise) without the possibility of changing the options, for instance, after seeing the collected data (i.e., exhaustive).

However, we agree that data-driven approaches to adjust, for instance, specific parameters necessary for a preprocessing step are important as those can differ between studies and depend on the data. For instance, parameter tuning for motion artifact detection might differ between participants, instruments, and brain regions.^133^ Accordingly, it is highly recommended to run a pilot study on which these decisions can be based.^7^ A pilot study can help to estimate the feasibility of the chosen parameters or algorithms, verify activation in ROIs, and test the pipeline for possible errors. Alternatively, certain preprocessing steps can be tested on similar data from existing open datasets, emphasizing the importance of data and code sharing (see section Sec. 2.6).

In the following sections, we focus on the main and, in most cases, absolute necessary preprocessing steps in CW-fNIRS research. We appreciate that there are other fNIRS systems (e.g., time domain NIRS and frequency domain NIRS), experimental setups, and designs that might require preprocessing steps that can differ from the ones mentioned here. However, by controlling whether the documentation of the steps is specific, precise, and exhaustive, finding the correct way of accurately documenting them should be straightforward.

##### Modified Beer–Lambert law

CW-fNIRS cannot directly measure absolute values of hemoglobin concentration due to its inability to determine the optical properties of the underlying tissues, for instance, the amount of absorbed and scattered light.^36^ Instead, it uses the modified Beer–Lambert law (mBLL) to calculate hemoglobin concentration changes, taking together the linear relationship between the optical density (i.e., how much light survives the path from the source to the detector) and the concentration of a medium and the assumption of constant light scattering.

However, the mBLL depends on several parameters that should be determined beforehand, such as the wavelength-dependent differential pathlength factors (DPFs) and the molar extinction coefficients. First, as DPFs, a fixed or an age-adjusted value can be chosen.^134^^,^^135^ Alternatively, a partial pathlength factor (PPF^134^^,^^135^), which corrects for the effective pathlength of the brain-relevant tissue instead of the pathlength through all tissues, can be used. If a default value of DPF/PPF is used, this should be mentioned accordingly (e.g., DPF = 6 as in the HomER2/HomER3 software^136^ or PPF = 0.1 as in the NIRS Brain AnalyzIR toolbox^137^ or HomER2/HomER3^136^^,^^137^). Second, tabularized molar extinction coefficients are typically applied in the mBLL. For more information about the mBLL, see for instance Refs. 36, 56, 134, 135, and 137.

##### Motion artifact correction

Motion artifacts in the fNIRS time courses result mostly from optode-scalp decoupling and/or head movement. These artifacts are typically characterized by spikes and baseline shifts in the fNIRS signal^133^ and, if not properly corrected, can highly decrease the reliability of the underlying hemodynamic response. There are several motion artifact correction algorithms available,^60^^,^^133^^,^^138^^,^^139^ and these can be split into two categories: (1) algorithms that rely on a prior motion artifact detection step and (2) algorithms that need no extra step for motion artifact detection. However, in both cases, most of the algorithms require parameter tuning.

Accordingly, in the case of (1), the motion artifact detection procedure and all respective parameters and thresholds should be reported (e.g., a motion artifact was defined as present if a signal exceeded the M±X times SD). For both (1) and (2), the correction method including all parameters and/or corresponding thresholds should be documented (e.g., a filter based on principal component analysis that accounted for 85% of the variance in the signal was applied). Further, whether the choice of thresholds is based on the literature or pilot data should be mentioned.

For possible choices of motion artifact algorithms, see for example Refs. 60, 125, 133, and 138–145.

##### Filtering

A major part of the noise in the fNIRS signal is related to non-evoked or spontaneous physiological processes.^36^^,^^126^ The frequency components mainly result from heartbeat (∼1  Hz), respiration (∼0.3  Hz), Mayer waves (∼0.1  Hz), and very low frequency oscillations (∼0.01 to 0.05 Hz).^9^^,^^36^ Depending on the task frequency, i.e., 1/(task period [s] + rest period [s]), some components can already relatively easily be eliminated by applying a conventional temporal filter.^9^ In the preregistration, whether or not a filter will be used should be noted.

As already recommended by Pinti et al.,^9^ whether an infinite or finite impulse response (i.e., infinite impulse response versus finite impulse response) filter will be applied should be documented, and the filter type itself should be specified (e.g., Butterworth filter and moving-average filter). Moreover, the filter design (e.g., low-pass, high-pass, or band-pass filter), cut-off frequencies (e.g., band-pass filter with cut-off frequencies of [0.01, 0.09] Hz) and the filter order (e.g., a second-order Butterworth filter) should be documented. And finally, it is important to specify whether a causal [e.g., MATLAB function *filter()*] or an acausal/zero-phase filter [e.g., MATLAB function *filtfilt()*] will be applied.

The process of digital filtering is a complex topic and can be deepened by considering for instance Refs. 146 and 147. For the specific application on fNIRS data we highly recommend Ref. 9.

##### Systemic activity correction

Other signal confounds might result from task-evoked cerebral and extracerebral systemic activity and are thus more difficult to remove. These artifacts mostly result from changes in the partial pressure of arterial carbon dioxide as well as blood pressure.^36^^,^^126^ They can mask or mimic hemodynamic activity and hence, if not properly corrected, can result in false negative or false positive results.^36^^,^^126^ Conventional temporal filters fall short of removing these artifacts because the artifact frequencies can overlap with the task frequency. So far, the most accurate way of removing the extracerebral part of such artifacts is using the hardware-based solution: short-distance channels (SDCs). SDCs are generated by placing the source and detector at a distance of <1  cm, and ideally at ∼0.8  cm for adults,^94^ to capture the hemodynamic activity from extracerebral tissue only.^36^^,^^126^^,^^148^^–^^152^ SDC signals can be used for correcting the regular distance channels, for instance, by applying a regression-based approach. If no SDCs are available, it is recommended to apply an alternative method to reduce the extracerebral systemic activity.^8^^,^^19^^,^^150^^,^^152^

In any case, the type of algorithm that will be applied for correction should be documented. For instance, for a simple regression, it is possible to use the closest SDC, the SDC with the highest correlation, the average across SDCs, or the first n principal components resulting from a principal component analysis of all SDCs. How correction will be handled if a number of SDCs have a poor channel quality should be further noted.

If no SDCs are available for correction, the algorithm and potential algorithm-specific parameters should be specified. For instance, if a global signal is computed (e.g., by taking the average of all channels), it is possible to simply subtract it from each channel or to use it as a regressor either for simple channel-wise regression or within a GLM framework. If a more advanced algorithm is applied, for instance, a principal component analysis of all available channels, the number of principal components (or the amount of explained variance) that are filtered out and the filter itself (e.g., subtracting, regression-based, and GLM) should be specified.

The topic of systemic activity correction is relatively new and very important for fNIRS preprocessing; hence, recently it has been much discussed, and methods have been validated in Refs. 36, 126, 148, and 150–154.

Preregistration template item(s): AP3 and AP4

### 2.4 Statistical Analysis

Similar to data preprocessing, the many options available for fNIRS statistics lead to analytic flexibility, and therefore any intended statistical test for confirmatory analysis should be preregistered before data analysis. We hereby differentiate between first-level (i.e., within-subject) and second-level (i.e., group) analyses. First-level analysis of fNIRS data is typically performed by block/trial averaging hemoglobin concentration changes over trials of the same condition or by modeling the signal response with a GLM.^9^^,^^35^^,^^155^ The advantage of GLM over block-averaging is its higher level of statistical power,^9^ and the possibility of modeling confounding factors along with the expected hemodynamic responses (e.g., by adding SDCs as nuisance regressors to the model).^126^ The choice of the GLM should be informed by the research context. For instance, if the shape of the expected HRF is unknown, then performing a block averaging or a GLM-based deconvolution with multiple Gaussian functions is potentially preferable to performing a GLM with a fixed HRF shape.^156^^–^^158^ For the second-level analysis, certain variables of the first-level analysis are chosen (e.g., mean of the block average over a certain time period, GLM estimates for certain conditions), and inferences are made with appropriate frequentist or Bayesian statistical tests (e.g., t-test, ANOVA, and mixed models). All chosen methods and variables should appropriately be reported and justified, and the researcher should clearly specify on which measured variable the research hypothesis is tested [e.g., oxygenated hemoglobin (HbO), deoxygenated hemoglobin (HbR), total hemoglobin, and hemoglobin difference]. Reporting of both HbO and HbR in the paper or its supplementary materials is recommended even if the primary hypotheses refer to only one measure.

**Fig. 4:**
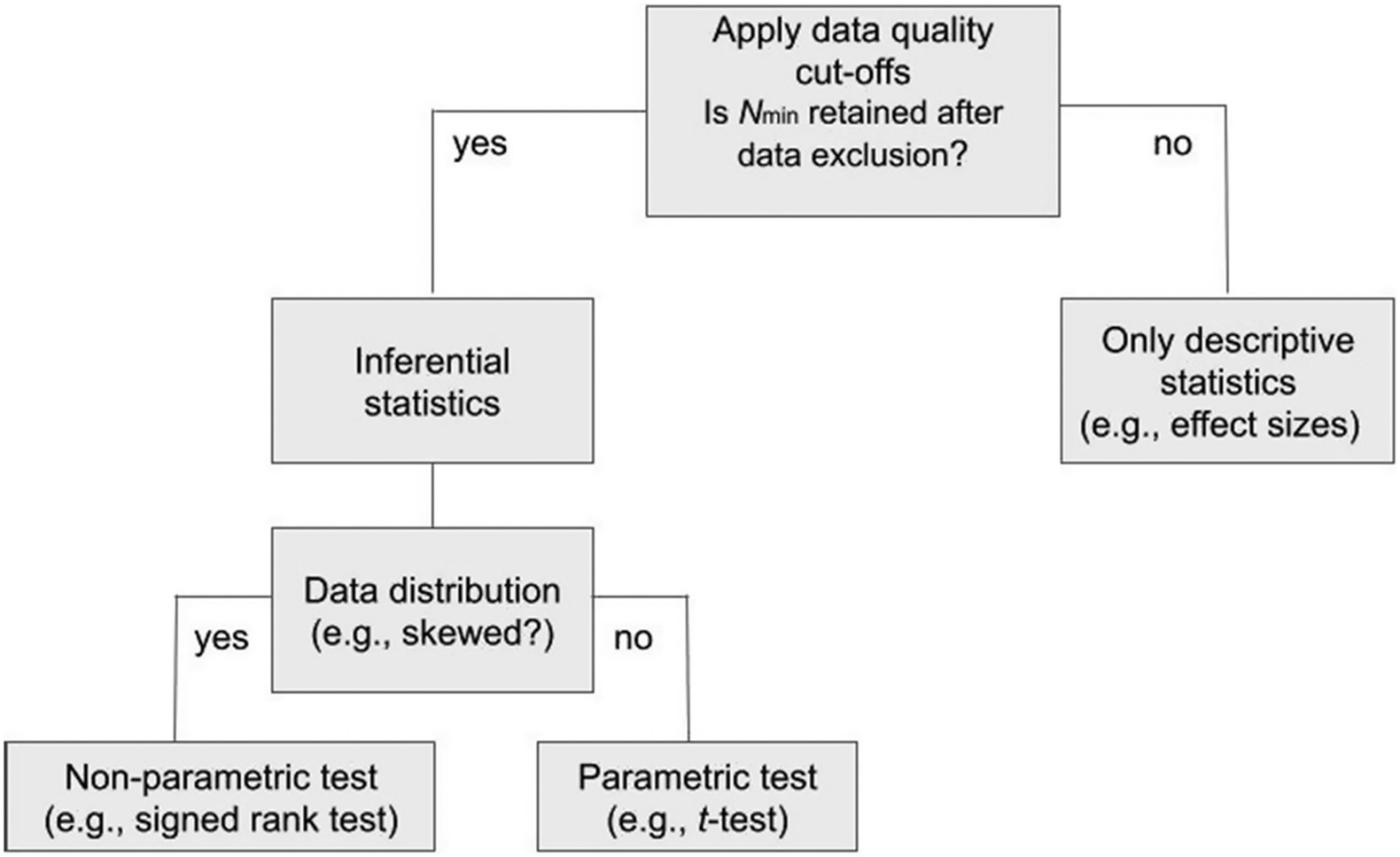
Example decision tree for fundamental test choices that are contingent on the data.

To control for researchers’ degrees of freedom, clear and comprehensible guidelines for analytic decisions are critical. For complex data with numerous interdependent preprocessing steps the use of analytic decision trees is encouraged (cf., Fig. 4). These define the sequence of analysis steps and decision rules that will be applied, depending on the outcome of a previous analysis step.^13^ For example, analytic decision trees may start with quality criteria thresholds, continue with assumption checks for statistical tests (e.g., parametric versus non-parametric), and follow with branches for adequate statistical tests, depending on whether respective test assumptions are met or not (cf., Fig. 4). Analytic decision trees can also be used to specify which follow-up tests are carried out and for the event of inconclusive findings. Results initially found to be inconclusive (often erroneously referred to as null findings) may still be informative if followed up with frequentist equivalence tests or Bayesian statistics that can provide evidence for the absence of an effect.^48^ In general, the results of any analysis should be reported in the final manuscript irrespective of their outcome.

Regardless of the choice to perform a block average or GLM, all potential analytical options related to the method should be mentioned in the preregistration. For instance, when performing a block average, the researcher should specify the variable that will be used for the second-level analysis (e.g., average over a certain time period, peak, time to peak, slope, area under the curve), as well as the way of defining this variable and the applied time periods (e.g., time period used for baseline correction and average over task period). In contrast, when choosing a GLM-based analysis, the researcher should clearly state not only the variable used for the second-level analysis (e.g., beta values) but also the details about the model: the specific HRF model used including its parameters (e.g., a double gamma function with $\tau_{\mathrm{HbO}}=0.1$; $\sigma_{\mathrm{HbO}}=3.0$; $\tau_{\mathrm{HbR}}=1.8$; and $\sigma_{\mathrm{HbR}}=3$),^159^ the extra nuisance regressors added to the model (e.g., all available HbO and HbR SDCs, accelerometers),^150^^,^^160^ and the method used to solve the GLM (e.g., ordinary least-squares and autoregressive iteratively reweighted least-squares model).^161^

Further, when multiple fNIRS channels and measures (e.g., HbO and HbR) are tested simultaneously, there is an increased risk of obtaining false positive results. When applying inferential frequentist statistics, the significance level should be declared (and ideally justified^162^^,^^163^). Furthermore, adequate correction methods for multiple comparisons should be stated (e.g., false discovery rate, Holm correction, and Bonferroni correction).^35^^,^^164^^–^^166^ To limit the number of statistical tests performed, researchers may first combine signals across multiple neighboring channels, yielding a smaller number of ROIs.^167^ However, this approach requires careful specification of the methods used to define (1) which channel belongs to which ROI and how was this defined (cf., Sec. 2.2.5 Optode array design and optode placement) and (2) how the data over channels belonging to the same ROI are combined (e.g., average, weighted average, the channel with the highest activity, and the channel with the highest sensitivity based on a localizer task). For Bayesian statistical approaches, the used prior distributions should be declared (and ideally be justified^168^). We further encourage researchers to report descriptive statistics and effect sizes, and where possible, to use effect sizes that are easy to interpret.^169^^,^^170^ For fNIRS studies that employ machine-learning methodologies including classifiers, we recommend consulting respective debates about best practices and the role of study preregistration.^171^^–^^176^

Preregistration template item(s): M13, AP5, AP6, and AP7

### 2.5 Optional: Exploratory Analyses

During or after data collection and analysis, new research questions and hypotheses may emerge. We refer to those as post-hoc exploratory analyses, which should be differentiated from planned exploratory analyses. Preregistration of an fNIRS study lends credibility to a researcher’s hypothesis-driven analysis plan and distinguishes planned confirmatory from post-hoc exploratory analyses. In contrast to confirmatory hypotheses and analyses, post-hoc exploratory analyses tend to investigate intuitions and ideas that evolve during the research process but did not primarily guide the research design. By this definition, exploratory analyses have lower prior odds, leading to a higher rate of false positive findings.^177^ Overall, unplanned and non-preregistered exploratory analyses have a characteristic that can inform unspecific theories.^178^ Reporting of unplanned and non-preregistered exploratory results might therefore potentially inspire subsequent confirmatory study designs. As such they should be given less weight in the overall conclusion of the study in which they were investigated and first replicated via independent, confirmatory testing. Therefore, exploratory analyses need to be labeled explicitly and differentiated from confirmatory analyses (although some authors argue for purely confirmatory scientific research^26^^,^^179^). However, researchers may have exploratory analyses already in mind at the planning stage, which they can preregister as planned exploratory analyses (i.e., either as specific analyses or as part of a decision tree under different analysis options/scenarios/outcomes; cf., Fig. 4), lending them more credibility compared with post-hoc (i.e., non-preregistered) exploratory analyses that may be subjected to undisclosed analytical flexibility and QRPs. Of note, preregistration does not preclude transparent, exhaustive post-hoc data exploitation in the form of multi-verse analyses that can be of particular methodological interest (e.g., artificial groups, moderators, and alternative data processing strategies^2^^,^^180^).

Preregistration template item(s): AP9

### 2.6 Data and Code Availability

Sharing data and code is undoubtedly part of open science. In general, open data and code improve verification and reproducibility of study results, increase citation rates, encourage data reuse for new research ideas, and are therefore increasingly part of research policies of public funders, ethical committees, and scientific journals.^181^^–^^183^ With regards to preregistration, we specifically encourage data and code sharing as a source for researchers new to fNIRS to plan their own analysis pipelines.

There are several reasons that data and code sharing should be considered prior to data collection. First, organizing and labeling data during data collection will save much time in the end when sharing data with other researchers in an understandable way. Second, analysis pipelines that work on sharable file formats and data structures will simplify the verification process for later reviewers. Third, sharing analysis code prior to data collection or keeping a version control can be considered the ultimate way of preregistration as it offers full transparency about changes in analysis plans. We specifically encourage this for the validation of new analysis methods as this should prevent overfitting of methodological choices based on the obtained research data. Finally, when drafting informed consent forms for future participants, researchers should consider asking permission to openly share personal data in accordance with local regulatory and institution specific data protection regulations.^184^

In general, shared data and code should be compliant with the FAIR principles (i.e., findable, accessible, interoperable, and reusable).^185^ Here, we provide some practical tips to make fNIRS data and code FAIR.

First, fNIRS data should be converted to .snirf^186^ (shared NIR spectroscopy format, https://github.com/fNIRS/snirf) files and organized and labeled according to the Brain Imaging Data Structure (BIDS).^185^^,^^187^ This standardization can help to increase interoperability and reusability, i.e., the data contains enough details to be easily interpreted by humans and machines. Specifically, .snirf is a standardized open access data format that is developed by the fNIRS community to facilitate sharing and analysis of fNIRS data, whereas BIDS is a standard specifying the organization and naming of data and metadata of neuroimaging studies. It is noteworthy that BIDS was recently extended (version 1.8.0) to include fNIRS data as a newly supported modality.^188^

Second, several platforms and repositories are available to enhance the findability and accessibility of data, code, and preregistrations. Data sharing repositories can vary from very general (e.g., Zenodo) to more specific for neuro-imaging (e.g., openneuro and NITRC) or even fNIRS data (e.g., openfnirs). When it comes to code sharing, platforms that include versioning control, such as GitHub, GitLab, or BitBucket, are preferred. Similarly, multiple options are available to share a preregistered study (e.g., on the OSF; osf.io). Further, the OSF provides a platform for organizing and version-controlled sharing data, code, and all other related research materials of one research project^185^^,^^187^^,^^189^ and provides the option to embargo a preregistration (i.e., it will only be made publicly available after a preset period of time). In Table 2, we provide an overview of several available data formats, repositories, and sharing platforms.

**Table 2:** Overview of available fNIRS-data standards and data-sharing repositories that aim to improve the FAIR-ness of research.

	Specialized for sharing of:	Description	Website
Standards			
.snirf	Raw data	Open-source file format for sharing fNIRS data.	https://github.com/fNIRS/snirf
BIDS	Raw data	Standard specifying the organization and naming of raw data and metadata of neuroimaging studies.	Refs. 190, 191
**Repositories**			
Openfnirs	Data	Platform sharing open-access fNIRS datasets.	Ref. 192
Openneuro	Data	Platform for validating and sharing BIDS-compliant neuroimaging datasets.	Ref. 193
NITRC-IR	Data	Image repository sharing neuroimaging datasets.	Ref. 194
brainlife	Data, code	Platform for sharing neuroscience code (apps) and data with an integrated cloud-computing and high-performance computing environment to run the apps.	Ref. 195
Zenodo	Data, code	General-purpose open repository developed by the European Commission for sharing data, code, papers, and reports.	https://zenodo.org/
GitHub	Code	Developer-focused platform to share code and software, including version control.	https://github.com/
GitLab	Code	Developer-focused platform to share code and software, including version control.	Ref. 196
BitBucket	Code	Platform to share code and software, including version control (private repositories for up to five users).	Ref. 197
OSF	Preregistration, data, code	Platform to organize and share all research data and material related to a research project, including version control; different preregistration templates, including open formats, can be used.	Ref. 198
Peer Community in Registered Reports	Registered Reports, preprints	Journal-independent research community platform that performs and publishes peer reviews of preprints as well as stage 1 and stage 2 reviews of Registered Reports. Following a positive evaluation of the complete review process, authors may submit their final Registered Report manuscript to cooperating journals.	Ref. 30
**Other useful platforms:**			
INCF		Portfolio with standards and best practices supporting open and FAIR neuroscience.	Ref. 199
FAIRsharing		Resource platform on data and metadata standards, inter-related on databases and data policies.	Ref. 200

Third, when researchers intend to share data with other researchers, they should carefully reflect on the balance between open science practices and privacy protection regulations,^201^^,^^202^ specifically the General Data Protection Regulation for research conducted in the European Union and the United Kingdom, the Health Insurance Portability and Accountability Act for research conducted in the United States of America, and similar regulations in other countries. As far as we know, the fNIRS time series signal itself is not identifiable and therefore, in principle, can be shared as anonymous data that does not fall under the privacy protection rules.^203^ However, data from participants is never measured solely by itself but often is linked to direct or indirect personal identifiers. For example, the name and contact information of participants (i.e., direct identifiers) are usually registered for administrative purposes. Researchers typically remove the direct link between the fNIRS data and these personal identifiers by pseudonymization. In addition, it is often desired and/or encouraged for scientific purposes to give context to our data with demographic and clinical information (Sec. 2.2.2). The combination of these snippets of information – each of which by itself is not identifying – does increase the chance of identification of study participants by means of a linkage attack (see for example, Ref. 204) and can therefore be considered to be indirect identifiable data.

Data sharing should always be in accordance with local data protection laws and requirements of the institutional review boards. Because common principles apply to many, we give a few practical recommendations regarding data from fNIRS studies. First, we recommend explaining to participants why it is intended to share data, what data will be shared, and how it will be shared. Even for anonymous data, it is best practice to always ask for explicit consent, and it is required for pseudonymize data. Importantly, sharing directly identifiable information (e.g., name, recording date, birthdate, and address) is prohibited under commonly used data protection regulation. Researchers should be aware that participants may use social media to share their whereabouts; researchers are strongly advised to prevent that any information shared by the participant outside their control—such as the post on social media “I participated in a study at the university this morning”—can be linked inadvertently to information that is intended to become later publicly available through data sharing by the researcher. Photos and videos can also be directly identifiable. Photogrammetry pictures should not be shared. Instead, we advise that only the extracted optode and anatomical landmark positions of the photogrammetry pictures are shared, or in case participants are shown on the picture, their face and appearance should not be recognizable (e.g., via blurring or removal). We further recommend avoiding sharing data that is not essential for the research question or follow-up analyses but has a high disclosure risk (e.g., an unusual finding). Furthermore, one should reduce the amount of detail when it comes to meta data.^205^ For example, instead of reporting a table with the exact ages of participants, a range can be reported instead. Finally, many departments and universities employ data stewards or data protection managers that can advise researchers on how to comply with local and national data sharing policies and implement FAIR data sharing principles.

Preregistration template item(s): T10 and T11

## 3 Summary

This step-by-step guide aims to assist researchers in both planning the design and drafting a preregistration protocol for task-related CW-fNIRS studies. In particular, fNIRS-specific design aspects such as optode placement and adequate sample size planning, as well as analytical aspects around the signal quality and data analysis, are elaborated. One focus is also set on using open research tools that facilitate study planning for hemodynamic studies and data sharing for fNIRS. Altogether, this guide thus complements recent efforts in providing best practice guidance for fNIRS researchers for careful study planning and addresses the call for tools that support researchers in preregistering their fNIRS study.^18^^,^^19^ It is further accompanied by a comprehensive preregistration template that includes 48 items, covering key aspects that are exemplified. These tools can aid in particular researchers new to the field in getting acquainted with the relevant steps and decisions of fNIRS study planning, and they can serve as tools for designing fNIRS studies transparently through the means of study preregistration.

## References

[1] Carp J., “On the plurality of (methodological) worlds: estimating the analytic flexibility of FMRI experiments,” Front. Neurosci. 6, 149 (2012).1662-453X 10.3389/fnins.2012.00149 23087605 PMC3468892

[2] Churchill N. W. et al., “Correction: an automated, adaptive framework for optimizing preprocessing pipelines in task-based functional MRI,” PLoS One 10(12), e0145594 (2015). POLNCL 1932-6203 10.1371/journal.pone.0145594 26678485 PMC4683087

[3] Poldrack R. A. et al., “Scanning the horizon: towards transparent and reproducible neuroimaging research,” Nat. Rev. Neurosci. 18(2), 115–126 (2017). NRNAAN 1471-003X 10.1038/nrn.2016.167 28053326 PMC6910649

[r4] Mehler D., “11 - The replication challenge: is brain imaging next?” in Casting Light on the Dark Side of Brain Imaging, Raz A. Thibault R. T., Eds., pp. 67–71, Academic Press (2019).

[r5] Botvinik-Nezer R. et al., “Variability in the analysis of a single neuroimaging dataset by many teams,” Nature 582(7810), 84–88 (2020). 10.1038/s41586-020-2314-9 32483374 PMC7771346

[6] Niso G. et al., “Good scientific practice in MEEG research: progress and perspectives,” Neuroimage 257, 119056 (2022). NEIMEF 1053-8119 10.1016/j.neuroimage.2022.119056 35283287 PMC11236277

[7] Paul M. Govaart G. H. Schettino A., “Making ERP research more transparent: guidelines for preregistration,” Int. J. Psychophysiol. 164, 52–63 (2021). IJPSEE 0167-8760 10.1016/j.ijpsycho.2021.02.016 33676957

[8] Pfeifer M. D. Scholkmann F. Labruyère R., “Signal processing in functional near-infrared spectroscopy (fNIRS): methodological differences lead to different statistical results,” Front. Hum. Neurosci. 11, 641 (2017). 10.3389/fnhum.2017.00641 29358912 PMC5766679

[9] Pinti P. et al., “Current status and issues regarding pre-processing of fNIRS neuroimaging data: an investigation of diverse signal filtering methods within a general linear model framework,” Front. Hum. Neurosci. 12, 505 (2018). 10.3389/fnhum.2018.00505 30687038 PMC6336925

[10] Simmons J. P. Nelson L. D. Simonsohn U., “False-positive psychology: undisclosed flexibility in data collection and analysis allows presenting anything as significant,” Psychol. Sci. 22(11), 1359–1366 (2011).1467-9280 10.1177/0956797611417632 22006061

[11] Niso G. et al., “Open and reproducible neuroimaging: from study inception to publication,” NeuroImage 263, 119623 (2022). 10.1016/j.neuroimage.2022.119623 36100172 PMC10008521

[r12] Lowndes J. S. S. et al., “Our path to better science in less time using open data science tools,” Nat. Ecol. Evol. 1(6), 160 (2017). 10.1038/s41559-017-0160 28812630

[r13] Nosek B. A. et al., “The preregistration revolution,” Proc. Natl. Acad. Sci. U. S. A. 115(11), 2600–2606 (2018). 10.1073/pnas.1708274114 29531091 PMC5856500

[14] Allen C. Mehler D. M. A., “Open science challenges, benefits and tips in early career and beyond,” PLoS Biol. 17(5), e3000246 (2019). 10.1371/journal.pbio.3000246 31042704 PMC6513108

[15] Nosek B. A. et al., “Replicability, robustness, and reproducibility in psychological science,” Annu. Rev. Psychol. 73, 719–748 (2022). ARPSAC 0066-4308 10.1146/annurev-psych-020821-114157 34665669

[16] Gorgolewski K. J. Poldrack R. A., “A practical guide for improving transparency and reproducibility in neuroimaging research,” PLoS Biol. 14(7), e1002506 (2016). 10.1371/journal.pbio.1002506 27389358 PMC4936733

[17] Gentili C. et al., “The case for preregistering all region of interest (ROI) analyses in neuroimaging research,” Eur. J. Neurosci. 53(2), 357–361 (2021). EJONEI 0953-816X 10.1111/ejn.14954 32852863

[18] Kelsey C. M. et al., “Shedding light on functional near infrared spectroscopy and open science practices,” https://www.biorxiv.org/content/10.1101/2022.05.13.491838v1 (2022). 10.1117/1.NPh.10.2.023520 PMC10109256 37077217

[r19] Yücel M. A. et al., “Best practices for fNIRS publications,” Neurophotonics 8(1), 012101 (2021). 10.1117/1.NPh.8.1.012101 33442557 PMC7793571

[20] Kohl S. H. et al., “The potential of functional near-infrared spectroscopy-based neurofeedback—a systematic review and recommendations for best practice,” Front. Neurosci. 14, 594 (2020).1662-453X 10.3389/fnins.2020.00594 32848528 PMC7396619

[21] Sarafoglou A. et al., “A survey on how preregistration affects the research workflow: better science but more work,” R. Soc. Open Sci. 9(7), 211997 (2022). 10.1098/rsos.211997 35814910 PMC9257590

[22] Nosek B. A. Lindsay D. S., “Preregistration becoming the norm in psychological science,” APS Obs. 31(3) (2018).

[23] Paret C. et al., “Survey on open science practices in functional neuroimaging,” Neuroimage 257, 119306 (2022). NEIMEF 1053-8119 10.1016/j.neuroimage.2022.119306 35595201

[24] Wicherts J. M. et al., “Degrees of freedom in planning, running, analyzing, and reporting psychological studies: a checklist to avoid p-hacking,” Front. Psychol. 7, 1832 (2016).1664-1078 10.3389/fpsyg.2016.01832 27933012 PMC5122713

[r25] Rubin M., “When does HARKing hurt? Identifying when different types of undisclosed post hoc hypothesizing harm scientific progress,” Rev. Gen. Psychol. 21(4), 308–320 (2017). 10.1037/gpr0000128

[r26] Wagenmakers E.-J. et al., “An agenda for purely confirmatory research,” Perspect. Psychol. Sci. 7(6), 632–638 (2012). 10.1177/1745691612463078 26168122

[r27] Nosek B. A. et al., “Preregistration is hard, and worthwhile,” Trends Cognit. Sci. 23(10), 815–818 (2019). 10.1016/j.tics.2019.07.009 31421987

[28] Hardwicke T. E. Wagenmakers E.-J., “Reducing bias, increasing transparency, and calibrating confidence with preregistration,” Nat. Hum. Behav. 7, 15–26 (2023). 10.1038/s41562-022-01497-2 36707644

[29] Chambers C. D. Tzavella L., “The past, present and future of Registered Reports,” Nat. Hum. Behav. 6(1), 29–42 (2022). 10.1038/s41562-021-01193-7 34782730

[30] Peer Community In, https://rr.peercommunityin.org/.

[31] Kvarven A. Strømland E. Johannesson M., “Author correction: comparing meta-analyses and preregistered multiple-laboratory replication projects,” Nat. Hum. Behav. 4(6), 659–663 (2020). 10.1038/s41562-020-0864-3 32488133

[32] Schäfer T. Schwarz M. A., “The meaningfulness of effect sizes in psychological research: differences between sub-disciplines and the impact of potential biases,” Front. Psychol. 10, 813 (2019).1664-1078 10.3389/fpsyg.2019.00813 31031679 PMC6470248

[33] Scheel A. M. Schijen M., “An excess of positive results: comparing the standard psychology literature with registered reports,” Adv. Methods and Practices in Psychological Sci. 4(2) (2021). 10.1177/25152459211007467

[34] Bosnjak M. et al., “A template for preregistration of quantitative research in psychology: report of the joint psychological societies preregistration task force,” Am. Psychol. 77, 602–615 (2021). AMPSAB 0003-066X 10.1037/amp0000879 34807636

[35] Tak S. Ye J. C., “Statistical analysis of fNIRS data: a comprehensive review,” NeuroImage 85, 72–91 (2014). 10.1016/j.neuroimage.2013.06.016 23774396

[36] Scholkmann F. et al., “A review on continuous wave functional near-infrared spectroscopy and imaging instrumentation and methodology,” Neuroimage 85(Pt 1), 6–27 (2014). NEIMEF 1053-8119 10.1016/j.neuroimage.2013.05.004 23684868

[37] Field S. M. et al., “The effect of preregistration on trust in empirical research findings: results of a registered report,” R. Soc. Open Sci. 7(4), 181351 (2020). 10.1098/rsos.181351 32431853 PMC7211853

[38] Song H. Markowitz D. M. Taylor S. H., “Trusting on the shoulders of open giants? Open science increases trust in science for the public and academics,” J. Commun. 72(4), 497–510 (2021). 10.1093/joc/jqac017

[39] Havron N. Bergmann C. Tsuji S., “Preregistration in infant research-a primer,” Infancy 25(5), 734–754 (2020). 10.1111/infa.12353 32857441

[r40] van den Akker O. R. et al., “Preregistration of secondary data analysis: a template and tutorial,” Mol. Pathol. 5, 2625 (2021). 10.15626/MP.2020.2625

[r41] Aczel B. et al., “A consensus-based transparency checklist,” Nat. Hum. Behav. 4(1), 4–6 (2020). 10.1038/s41562-019-0772-6 31792401 PMC8324470

[r42] Beyer F. et al., “A fMRI pre-registration template,” PsychArchives (2021).

[r43] Ros T. et al., “Consensus on the reporting and experimental design of clinical and cognitive-behavioural neurofeedback studies (CRED-nf checklist),” Brain 143(6), 1674–1685 (2020). BRAIAK 0006-8950 10.1093/brain/awaa009 32176800 PMC7296848

[r44] Crüwell S. Evans N. J., “Preregistration in diverse contexts: a preregistration template for the application of cognitive models,” R. Soc. Open Sci. 8(10), 210155 (2021). 10.1098/rsos.210155 34659776 PMC8511762

[45] Govaart G. H. et al., “EEG ERP preregistration template,” 2022, https://osf.io; metaarxivhttps://osf.io; metaarxivhttps://osf.io; pvrn6https://osf.io, pvrn6.

[46] https://uni-tuebingen.de/de/202162.

[47] Dirnagl U., “Preregistration of exploratory research: learning from the golden age of discovery,” PLoS Biol. 18(3), e3000690 (2020). 10.1371/journal.pbio.3000690 32214315 PMC7098547

[r48] Mehler D. M. A. Edelsbrunner P. A. Matić K., “Appreciating the significance of non-significant findings in psychology,” J. Eur. Psychol. Stud. 10(4), 1–7 (2019). 10.5334/e2019a

[r49] Scheel A. M. et al., “Why hypothesis testers should spend less time testing hypotheses,” Perspect. Psychol. Sci. 16(4), 744–755 (2021). 10.1177/1745691620966795 33326363 PMC8273364

[r50] Dorta-González P. González-Betancor S. M. Dorta-González M. I., “To what extent is researchers’ data-sharing motivated by formal mechanisms of recognition and credit?” Scientometrics 126(3), 2209–2225 (2021). SCNTDX 0138-9130 10.1007/s11192-021-03869-3

[r51] Crüwell S. et al., “Seven easy steps to open science: an annotated reading list,” Zeitschr. Psychol. 227(4), 237–248 (2019). 10.1027/2151-2604/a000387

[r52] UK Reproducibility Network Steering Committee, “From grassroots to global: a blueprint for building a reproducibility network,” PLoS Biol. 19(11), e3001461 (2021). 10.1371/journal.pbio.3001461 34758016 PMC8608297

[r53] Rahal R.-M. et al., “The German reproducibility network – a strategic community effort to promote transparent research practices in the scientific system,” presented at Open Sci. Conf. (2021).

[54] Bielczyk N. Z. et al., “Effective self-management for early career researchers in the natural and life sciences,” Neuron 106(2), 212–217 (2020). NERNET 0896-6273 10.1016/j.neuron.2020.03.015 32325057 PMC7665085

[55] https://osf.io/hb4um/.

[56] Pinti P. et al., “The present and future use of functional near-infrared spectroscopy (fNIRS) for cognitive neuroscience,” Ann. N. Y. Acad. Sci. 1464(1), 5–29 (2020). ANYAA9 0077-8923 10.1111/nyas.13948 30085354 PMC6367070

[57] Baldwin J. R. et al., “Protecting against researcher bias in secondary data analysis: challenges and potential solutions,” Eur. J. Epidemiol. 37(1), 1–10 (2022). EJEPE8 10.1007/s10654-021-00839-0 35025022 PMC8791887

[58] Gusnard D. A. Raichle M. E. Raichle M. E., “Searching for a baseline: functional imaging and the resting human brain,” Nat. Rev. Neurosci. 2(10), 685–694 (2001). 10.1038/35094500 11584306

[59] Piazza C. et al., “Preprocessing pipeline for fNIRS data in children,” in XV Mediterranean Conf. Med. and Biol. Eng. and Comput.—MEDICON 2019, Springer International Publishing, pp. 235–244 (2020).

[60] Di Lorenzo R. et al., “Recommendations for motion correction of infant fNIRS data applicable to multiple data sets and acquisition systems,” Neuroimage 200, 511–527 (2019). NEIMEF 1053-8119 10.1016/j.neuroimage.2019.06.056 31247300

[61] Parker T. C. Ricard J. A., “Structural racism in neuroimaging: perspectives and solutions,” Lancet Psychiatr. 9(5), e22 (2022). 10.1016/S2215-0366(22)00079-7 35430005

[r62] Sjoding M. W. et al., “Racial bias in pulse oximetry measurement,” N. Engl. J. Med. 383(25), 2477–2478 (2020). NEJMAG 0028-4793 10.1056/NEJMc2029240 33326721 PMC7808260

[r63] Wassenaar E. B. Van den Brand J. G. H., “Reliability of near-infrared spectroscopy in people with dark skin pigmentation,” J. Clin. Monit. Comput. 19(3), 195–199 (2005). 10.1007/s10877-005-1655-0 16244841

[64] Afshari A. et al., “Evaluation of the robustness of cerebral oximetry to variations in skin pigmentation using a tissue-simulating phantom,” Biomed. Opt. Express 13(5), 2909–2928 (2022). BOEICL 2156-7085 10.1364/BOE.454020 35774336 PMC9203096

[65] Lakens D., “Sample size justification,” Collabra Psychol. 8(1), 33267 (2022). 10.1525/collabra.33267

[66] Field A. Miles J. Field Z., Discovering Statistics Using R, Sage, London (2012).

[67] Albers C. Lakens D., “When power analyses based on pilot data are biased: Inaccurate effect size estimators and follow-up bias,” J. Exp. Soc. Psychol. 74, 187–195 (2018). JESPAQ 0022-1031 10.1016/j.jesp.2017.09.004

[68] Algermissen J. Mehler D. M. A., “May the power be with you: are there highly powered studies in neuroscience, and how can we get more of them?” J Neurophysiol 119(6), 2114–2117 (2018). JONEA4 0022-3077 10.1152/jn.00765.2017 29465324

[69] Schönbrodt F. D. Wagenmakers E.-J., “Bayes factor design analysis: planning for compelling evidence,” Psychon. Bull. Rev. 25(1), 128–142 (2018). PBUREN 1069-9384 10.3758/s13423-017-1230-y 28251595

[70] Stefan A. M. et al., “A tutorial on Bayes factor design analysis using an informed prior,” Behav. Res. Methods 51(3), 1042–1058 (2019). 10.3758/s13428-018-01189-8 30719688 PMC6538819

[71] Boayue N. M. et al., “Increasing propensity to mind-wander by transcranial direct current stimulation? A registered report,” Eur. J. Neurosci. 51(3), 755–780 (2020). EJONEI 0953-816X 10.1111/ejn.14347 30680810

[72] Mehler D. M. A. et al., “Graded fMRI neurofeedback training of motor imagery in middle cerebral artery stroke patients: a preregistered proof-of-concept study,” Front. Hum. Neurosci. 14, 226 (2020). 10.3389/fnhum.2020.00226 32760259 PMC7373077

[73] Lakens D. Caldwell A. R., “Simulation-based power analysis for factorial analysis of variance designs,” Adv. Methods Pract. Psychol. Sci. 4(1), 251524592095150 (2021). 10.1177/2515245920951503

[74] https://shiny.ieis.tue.nl/anova_power/.

[75] https://brainpower.readthedocs.io/en/latest/index.html#.

[76] https://aaroncaldwell.us/SuperpowerBook/.

[77] The Jamovi Project, “jamovi (Version 1.6) [Computer Software],” Computer Software (2020).

[78] https://www.jamovi.org/.

[79] Faul F. et al., “G*Power 3: a flexible statistical power analysis program for the social, behavioral, and biomedical sciences,” Behav. Res. Methods 39(2), 175–191 (2007). 10.3758/BF03193146 17695343

[80] Faul F. et al., “Statistical power analyses using G*Power 3.1: tests for correlation and regression analyses,” Behav. Res. Methods 41(4), 1149–1160 (2009). 10.3758/BRM.41.4.1149 19897823

[81] https://www.psychologie.hhu.de/arbeitsgruppen/allgemeine-psychologie-und-arbeitspsychologie/gpower.

[82] https://cran.r-project.org/web/packages/pwr/index.html.

[83] https://wiki.usask.ca/pages/viewpageattachments.action?pageId=420413544.

[84] Kovacs M. et al., “SampleSizePlanner: a tool to estimate and justify sample size for two-group studies,” Adv. Methods Pract. Psychol. Sci. 5(1), 251524592110540 (2022). 10.1177/25152459211054059

[85] Kovacs et al., “Sample size planner,” https://martonbalazskovacs.shinyapps.io/SampleSizePlanner/ (2021).

[86] Bartlett J. E. Charles S. J., Power to the People: A Beginner’s Tutorial to Power Analysis using jamovi (2021).

[87] Sun B. et al., “Behavioral and brain synchronization differences between expert and novice teachers when collaborating with students,” Brain Cognit. 139, 105513 (2020). 10.1016/j.bandc.2019.105513 31887711

[88] Brigadoi S. et al., “On pacing trials while scanning brain hemodynamics: the case of the SNARC effect,” Psychon. Bull. Rev. 25(6), 2267–2273 (2018). PBUREN 1069-9384 10.3758/s13423-017-1418-1 29340998

[89] Bartoš F. Maier M. Wagenmakers E. J., “Adjusting for publication bias in JASP—Selection models and robust Bayesian meta-analysis,” Adv. Methods and Practices in Psychological Sci. 5(3) (2022). 10.1177/25152459221109259

[90] Cuijpers P. et al., “What is the threshold for a clinically relevant effect? The case of major depressive disorders,” Depress. Anxiety 31(5), 374–378 (2014). 10.1002/da.22249 24677535

[r91] Lakens D. Scheel A. M. Isager P. M., “Equivalence testing for psychological research: a tutorial,” Adv. Methods Pract. Psychol. Sci. 1(2), 259–269 (2018). 10.1177/2515245918770963

[92] Lakens D. et al., “Improving inferences about null effects with Bayes factors and equivalence tests,” J. Gerontol. B Psychol. Sci. Soc. Sci. 75(1), 45–57 (2020). 10.1093/geronb/gby065 29878211

[93] Gerchen M. F. Kirsch P. Feld G. B., “Brain-wide inferiority and equivalence tests in fMRI group analyses: Selected applications,” Hum. Brain Mapp. 42(18), 5803–5813 (2021). HBRME7 1065-9471 10.1002/hbm.25664 34529303 PMC8596945

[94] Brigadoi S. Cooper R. J., “How short is short? Optimum source-detector distance for short-separation channels in functional near-infrared spectroscopy,” Neurophotonics 2(2), 025005 (2015). 10.1117/1.NPh.2.2.025005 26158009 PMC4478880

[95] Pinti P. et al., “A review on the use of wearable functional near-infrared spectroscopy in naturalistic environments,” Jpn. Psychol. Res. 60(4), 347–373 (2018). 10.1111/jpr.12206 30643322 PMC6329605

[96] Oostenveld R. Praamstra P., “The five percent electrode system for high-resolution EEG and ERP measurements,” Clin. Neurophysiol. 112(4), 713–719 (2001). CNEUFU 1388-2457 10.1016/S1388-2457(00)00527-7 11275545

[97] Jasper H. H., “Formal discussion: dendrites,” Electroencephalogr. Clin. Neurophysiol. Suppl. 35(Supp 10), 42–50 (1958). EECSB3 0424-8155 13609539

[98] Tsuzuki D. Dan I., “Spatial registration for functional near-infrared spectroscopy: from channel position on the scalp to cortical location in individual and group analyses,” Neuroimage 85(Pt 1), 92–103 (2014). NEIMEF 1053-8119 10.1016/j.neuroimage.2013.07.025 23891905

[99] Okamoto M. et al., “Three-dimensional probabilistic anatomical cranio-cerebral correlation via the international 10-20 system oriented for transcranial functional brain mapping,” Neuroimage 21(1), 99–111 (2004). NEIMEF 1053-8119 10.1016/j.neuroimage.2003.08.026 14741647

[100] Frijia E. M. et al., “Functional imaging of the developing brain with wearable high-density diffuse optical tomography: a new benchmark for infant neuroimaging outside the scanner environment,” NeuroImage 225, 117490 (2021). NEIMEF 1053-8119 10.1016/j.neuroimage.2020.117490 33157266

[r101] Mazzonetto I. et al., “Smartphone-based photogrammetry provides improved localization and registration of scalp-mounted neuroimaging sensors,” Sci. Rep. 12, 10862 (2022). SRCEC3 2045-2322 10.1038/s41598-022-14458-6 35760834 PMC9237074

[102] Jaffe-Dax S. et al., “Video-based motion-resilient reconstruction of three-dimensional position for functional near-infrared spectroscopy and electroencephalography head mounted probes,” Neurophotonics 7(3), 035001 (2020). 10.1117/1.NPh.7.3.035001 32704521 PMC7370942

[103] Benitez-Andonegui A. et al., “Guiding functional near-infrared spectroscopy optode-layout design using individual (f)MRI data: effects on signal strength,” Neurophotonics 8(2), 025012 (2021). 10.1117/1.NPh.8.2.025012 34155480 PMC8211086

[r104] Klein F. et al., “fMRI-based validation of continuous-wave fNIRS of supplementary motor area activation during motor execution and motor imagery,” Sci. Rep. 12(1), 3570 (2022). SRCEC3 2045-2322 10.1038/s41598-022-06519-7 35246563 PMC8897516

[105] Wu S. T. et al., “Accurate image-guided (Re)Placement of NIRS probes,” Comput. Methods Prog. Biomed. 200, 105844 (2021). CMPBEK 0169-2607 10.1016/j.cmpb.2020.105844 33267972

[106] Aasted C. M. et al., “Anatomical guidance for functional near-infrared spectroscopy: AtlasViewer tutorial,” Neurophotonics 2(2), 020801 (2015). 10.1117/1.NPh.2.2.020801 26157991 PMC4478785

[107] Brigadoi S. et al., “Array designer: automated optimized array design for functional near-infrared spectroscopy,” Neurophotonics 5(3), 035010 (2018). 10.1117/1.NPh.5.3.035010 30238021 PMC6135986

[108] Zimeo Morais G. A. Balardin J. B. Sato J. R., “fNIRS Optodes’ Location Decider (fOLD): a toolbox for probe arrangement guided by brain regions-of-interest,” Sci. Rep. 8(1), 3341 (2018). SRCEC3 2045-2322 10.1038/s41598-018-21716-z 29463928 PMC5820343

[109] Fu X. Richards J. E., “devfOLD: a toolbox for designing age-specific fNIRS channel placement,” Neurophotonics 8(4), 045003 (2021). 10.1117/1.NPh.8.4.045003 34881349 PMC8647945

[110] Vanegas M. Mireles M. Fang Q., “MOCA: a systematic toolbox for designing and assessing modular functional near-infrared brain imaging probes,” Neurophotonics 9(1), 017801 (2022). 10.1117/1.NPh.9.1.017801 36278785 PMC8823693

[111] Erel Y. et al., “STORM-Net: simple and timely optode registration method for functional near-infrared spectroscopy (fNIRS),” https://www.biorxiv.org/content/10.1101/2020.12.29.424683v3 (2021).

[112] Tzourio-Mazoyer N. et al., “Automated anatomical labeling of activations in SPM using a macroscopic anatomical parcellation of the MNI MRI single-subject brain,” Neuroimage 15(1), 273–289 (2002). NEIMEF 1053-8119 10.1006/nimg.2001.0978 11771995

[113] Rolls E. T. Joliot M. Tzourio-Mazoyer N., “Implementation of a new parcellation of the orbitofrontal cortex in the automated anatomical labeling atlas,” Neuroimage 122, 1–5 (2015). NEIMEF 1053-8119 10.1016/j.neuroimage.2015.07.075 26241684

[114] Rorden C. Brett M., “Stereotaxic display of brain lesions,” Behav. Neurol. 12(4), 191–200 (2000). BNEUEI 10.1155/2000/421719 11568431

[115] Holmes C. J. et al., “Enhancement of T1 MR images using registration for signal averaging,” Neuroimage 3(3), S28 (1996). NEIMEF 1053-8119 10.1016/S1053-8119(96)80030-9 9530404

[116] Penny W. D. et al., Statistical Parametric Mapping: The Analysis of Functional Brain Images, Elsevier (2011).

[117] Pollonini L. et al., “Auditory cortex activation to natural speech and simulated cochlear implant speech measured with functional near-infrared spectroscopy,” Hear. Res. 309, 84–93 (2014). HERED3 0378-5955 10.1016/j.heares.2013.11.007 24342740 PMC3939048

[118] Pollonini L. Bortfeld H. Oghalai J. S., “PHOEBE: a method for real time mapping of optodes-scalp coupling in functional near-infrared spectroscopy,” Biomed. Opt. Express 7(12), 5104–5119 (2016). BOEICL 2156-7085 10.1364/BOE.7.005104 28018728 PMC5175555

[119] Piper S. K. et al., “A wearable multi-channel fNIRS system for brain imaging in freely moving subjects,” Neuroimage 85(Pt 1), 64–71 (2014). NEIMEF 1053-8119 10.1016/j.neuroimage.2013.06.062 23810973 PMC3859838

[120] Selb J. et al., “Improved sensitivity to cerebral hemodynamics during brain activation with a time-gated optical system: analytical model and experimental validation,” J Biomed Opt 10(1), 011013 (2005). JBOPFO 1083-3668 10.1117/1.1852553 15847579

[121] Anderson T., “fNIRS MCT,” Study preregistration protocol, Open Science Framework, https://osf.io/5dftm (2021).

[122] Olson H., “Prefrontal cortical activity during learning in infants: an fNIRS study,” Study preregistration protocol. Open Science Framework, https://osf.io/nqpcs (2021).

[123] Fiske A., “Neural correlates of inhibitory control in 10-month-old infants: a functional near-infrared spectroscopy study,” Neuroimage 257, 119241 (2022). 10.1016/j.neuroimage.2022.119241 35537598 PMC7616317

[124] Artemenko C., “Developmental fronto-parietal shift of brain activation during mental arithmetic across the lifespan: a registered report protocol,” PLoS One 16(8), e0256232 (2021). POLNCL 1932-6203 10.1371/journal.pone.0256232 34432831 PMC8386861

[125] von Lühmann A. et al., “A new blind source separation framework for signal analysis and artifact rejection in functional near-infrared spectroscopy,” Neuroimage 200, 72–88 (2019). NEIMEF 1053-8119 10.1016/j.neuroimage.2019.06.021 31203024

[126] Tachtsidis I. Scholkmann F., “False positives and false negatives in functional near-infrared spectroscopy: issues, challenges, and the way forward,” Neurophotonics 3(3), 031405 (2016). 10.1117/1.NPh.3.3.031405 27054143 PMC4791590

[127] Hocke L. M. et al., “Automated processing of fNIRS data-a visual guide to the pitfalls and consequences,” Algorithms 11(5), 67 (2018).1748-7188 10.3390/a11050067 30906511 PMC6428450

[128] Klein F. Kranczioch C., “Signal processing in fNIRS: a case for the removal of systemic activity for single trial data,” Front. Hum. Neurosci. 13, 331 (2019). 10.3389/fnhum.2019.00331 31607880 PMC6769087

[129] Almajidy R. K. et al., “A newcomer’s guide to functional near infrared spectroscopy experiments,” IEEE Rev. Biomed. Eng. 13, 292–308 (2020). 10.1109/RBME.2019.2944351 31634142

[r130] “Software,” The Society for functional Near Infrared Spectroscopy, 2012, https://fnirs.org/resources/data-analysis/software/ (accessed 26 June 2022).

[131] Artinis Medical Systems, “fNIRS analysis toolbox series – Introduction — Artinis Medical Systems,” Artinis Medical Systems | fNIRS and NIRS devices, 2021, https://www.artinis.com/blogpost-all/2021/fnirs-analysis-toolboxes-introduction (accessed 26 June 2022).

[132] Cater B. T. Luke S. G., “Best practices in eye tracking research,” Int. J. Psychophysiol. 155, 49–62 (2020). IJPSEE 0167-8760 10.1016/j.ijpsycho.2020.05.010 32504653

[133] Fishburn F. A. et al., “Temporal derivative distribution repair (TDDR): a motion correction method for fNIRS,” Neuroimage 184, 171–179 (2019). NEIMEF 1053-8119 10.1016/j.neuroimage.2018.09.025 30217544 PMC6230489

[134] Scholkmann F. Wolf M., “General equation for the differential pathlength factor of the frontal human head depending on wavelength and age,” J. Biomed. Opt. 18(10), 105004 (2013). JBOPFO 1083-3668 10.1117/1.JBO.18.10.105004 24121731

[135] Whiteman A. C. et al., “Investigation of the sensitivity of functional near-infrared spectroscopy brain imaging to anatomical variations in 5- to 11-year-old children,” Neurophotonics 5(1), 011009 (2018). 10.1117/1.NPh.5.1.011009 28948192 PMC5601503

[136] Huppert T. J. et al., “HomER: a review of time-series analysis methods for near-infrared spectroscopy of the brain,” Appl. Opt. 48(10), D280–D298 (2009). APOPAI 0003-6935 10.1364/AO.48.00D280 19340120 PMC2761652

[137] Santosa H. et al., “The NIRS brain AnalyzIR toolbox,” Algorithms 11(5), 73 (2018).1748-7188 10.3390/a11050073 PMC11218834 38957522

[138] Cooper R. et al., “A systematic comparison of motion artifact correction techniques for functional near-infrared spectroscopy,” Front. Neurosci. 6, 147 (2012).1662-453X 10.3389/fnins.2012.00147 23087603 PMC3468891

[139] Brigadoi S. et al., “Motion artifacts in functional near-infrared spectroscopy: a comparison of motion correction techniques applied to real cognitive data,” Neuroimage 85(Pt 1), 181–191 (2014). NEIMEF 1053-8119 10.1016/j.neuroimage.2013.04.082 23639260 PMC3762942

[140] Scholkmann F. et al., “How to detect and reduce movement artifacts in near-infrared imaging using moving standard deviation and spline interpolation,” Physiol. Meas. 31(5), 649–662 (2010). PMEAE3 0967-3334 10.1088/0967-3334/31/5/004 20308772

[141] Novi S. L. et al., “Functional near-infrared spectroscopy for speech protocols: characterization of motion artifacts and guidelines for improving data analysis,” Neurophotonics 7(1), 015001 (2020). 10.1117/1.NPh.7.1.015001 31956662 PMC6953699

[142] Gemignani J. Gervain J., “Comparing different pre-processing routines for infant fNIRS data,” Dev. Cognit. Neurosci. 48, 100943 (2021). 10.1016/j.dcn.2021.100943 33735718 PMC7985709

[143] Delgado Reyes L. M. et al., “Evaluating motion processing algorithms for use with functional near-infrared spectroscopy data from young children,” Neurophotonics 5(2), 025008 (2018). 10.1117/1.NPh.5.2.025008 29845087 PMC5963607

[144] Huang R. et al., “Motion artifacts removal and evaluation techniques for functional near-infrared spectroscopy signals: a review,” Front. Neurosci. 16, 878750 (2022).1662-453X 10.3389/fnins.2022.878750 36263362 PMC9576156

[145] Behrendt H. F. et al., “Motion correction for infant functional near-infrared spectroscopy with an application to live interaction data,” Neurophotonics 5(1), 015004 (2018). 10.1117/1.NPh.5.1.015004 29487875 PMC5811207

[146] Smith S., Digital Signal Processing: A Practical Guide for Engineers and Scientists, Elsevier (2013).

[147] de Cheveigné A. Nelken I., “Filters: when, why, and how (not) to use them,” Neuron 102(2), 280–293 (2019). NERNET 0896-6273 10.1016/j.neuron.2019.02.039 30998899

[148] Wyser D. et al., “Short-channel regression in functional near-infrared spectroscopy is more effective when considering heterogeneous scalp hemodynamics,” Neurophotonics 7(3), 035011 (2020). 10.1117/1.NPh.7.3.035011 33029548 PMC7523733

[r149] Wyser D. G. et al., “Characterizing reproducibility of cerebral hemodynamic responses when applying short-channel regression in functional near-infrared spectroscopy,” Neurophotonics 9(1), 015004 (2022). 10.1117/1.NPh.9.1.015004 35265732 PMC8901194

[r150] Santosa H. et al., “Quantitative comparison of correction techniques for removing systemic physiological signal in functional near-infrared spectroscopy studies,” Neurophotonics 7(3), 035009 (2020). 10.1117/1.NPh.7.3.035009 32995361 PMC7511246

[r151] Abdalmalak A. et al., “Effects of systemic physiology on mapping resting-state networks using functional near-infrared spectroscopy,” Front. Neurosci. 16, 803297 (2022). 10.3389/fnins.2022.803297 35350556 PMC8957952

[152] Klein F. et al., “Performance comparison of systemic activity correction in functional near-infrared spectroscopy for methods with and without short distance channels,” Neurophotonics 10(1), 013503 (2023). 10.1117/1.NPh.10.1.013503 36248616 PMC9555616

[153] Kirilina E. et al., “The physiological origin of task-evoked systemic artefacts in functional near infrared spectroscopy,” Neuroimage 61(1), 70–81 (2012). NEIMEF 1053-8119 10.1016/j.neuroimage.2012.02.074 22426347 PMC3348501

[154] Scholkmann F. et al., “Systemic physiology augmented functional near-infrared spectroscopy: a powerful approach to study the embodied human brain,” Neurophotonics 9(3), 030801 (2022). 10.1117/1.NPh.9.3.030801 35832785 PMC9272976

[155] Luke R. et al., “Analysis methods for measuring passive auditory fNIRS responses generated by a block-design paradigm,” Neurophotonics 8(2), 025008 (2021). 10.1117/1.NPh.8.2.025008 34036117 PMC8140612

[156] Santosa H. et al., “Investigation of the sensitivity-specificity of canonical- and deconvolution-based linear models in evoked functional near-infrared spectroscopy,” Neurophotonics 6(2), 025009 (2019). 10.1117/1.NPh.6.2.025009 31172019 PMC6541797

[r157] Ciftçi K. et al., “Constraining the general linear model for sensible hemodynamic response function waveforms,” Med. Biol. Eng. Comput. 46(8), 779–787 (2008). MBECDY 0140-0118 10.1007/s11517-008-0347-6 18427851

[158] Lindquist M. A. et al., “Modeling the hemodynamic response function in fMRI: efficiency, bias and mismodeling,” Neuroimage 45(1), S187–S198 (2009). NEIMEF 1053-8119 10.1016/j.neuroimage.2008.10.065 19084070 PMC3318970

[159] Kamran M. A. Mannan M. M. N. Jeong M. Y., “Cortical signal analysis and advances in functional near-infrared spectroscopy signal: a review,” Front. Hum. Neurosci. 10, 261 (2016). 10.3389/fnhum.2016.00261 27375458 PMC4899446

[160] von Lühmann A. et al., “Improved physiological noise regression in fNIRS: a multimodal extension of the general linear model using temporally embedded Canonical Correlation Analysis,” Neuroimage 208, 116472 (2020). NEIMEF 1053-8119 10.1016/j.neuroimage.2019.116472 31870944 PMC7703677

[161] Barker J. W. Aarabi A. Huppert T. J., “Autoregressive model based algorithm for correcting motion and serially correlated errors in fNIRS,” Biomed. Opt. Express 4(8), 1366–1379 (2013). BOEICL 2156-7085 10.1364/BOE.4.001366 24009999 PMC3756568

[162] Maier M. Lakens D., “Justify your alpha: a primer on two practical approaches,” Adv. Methods and Practices in Psychological Sci. 5(2) (2022). 10.1177/25152459221080396

[163] Lakens D. et al., “Justify your alpha,” Nat. Hum. Behav. 2(3), 168–171 (2018). 10.1038/s41562-018-0311-x

[164] Singh A. K. Dan I., “Exploring the false discovery rate in multichannel NIRS,” Neuroimage 33(2), 542–549 (2006). NEIMEF 1053-8119 10.1016/j.neuroimage.2006.06.047 16959498

[r165] Plichta M. M. et al., “Event-related functional near-infrared spectroscopy (fNIRS): are the measurements reliable?” Neuroimage 31(1), 116–124 (2006). NEIMEF 1053-8119 10.1016/j.neuroimage.2005.12.008 16446104

[166] Benjamini Y. Hochberg Y., “Controlling the false discovery rate: a practical and powerful approach to multiple testing,” J. R. Stat. Soc. 57(1), 289–300 (1995).0952-8385 10.1111/j.2517-6161.1995.tb02031.x

[167] Okamoto M. et al., “Structural atlas-based spatial registration for functional near-infrared spectroscopy enabling inter-study data integration,” Clin. Neurophysiol. 120(7), 1320–1328 (2009). CNEUFU 1388-2457 10.1016/j.clinph.2009.01.023 19464945

[168] Kass R. E. Wasserman L., “The selection of prior distributions by formal rules,” J. Am. Stat. Assoc. 91(435), 1343–1370 (1996). 10.1080/01621459.1996.10477003

[169] Lakens D., “Calculating and reporting effect sizes to facilitate cumulative science: a practical primer for t-tests and ANOVAs,” Front. Psychol. 4, 863 (2013).1664-1078 10.3389/fpsyg.2013.00863 24324449 PMC3840331

[170] Hanel P. H. Mehler D. M., “Beyond reporting statistical significance: Identifying informative effect sizes to improve scientific communication,” Public Understanding Sci. 28(4), 468–485 (2019). PUNSEM 0963-6625 10.1177/0963662519834193 30848696

[171] Mongan J. Moy L. Kahn C. E. Jr., “Checklist for artificial intelligence in medical imaging (CLAIM): a guide for authors and reviewers,” Radiol. Artif. Intell. 2(2), e200029 (2020). 10.1148/ryai.2020200029 33937821 PMC8017414

[r172] Naseer N. Hong K.-S., “fNIRS-based brain-computer interfaces: a review,” Front. Hum. Neurosci. 9, 3 (2015). 10.3389/fnhum.2015.00003 25674060 PMC4309034

[r173] Leenings R. et al., “Recommendations for machine learning benchmarks in neuroimaging,” Neuroimage 257, 119298 (2022). NEIMEF 1053-8119 10.1016/j.neuroimage.2022.119298 35561945

[r174] Dukart J. et al., “Towards increasing the clinical applicability of machine learning biomarkers in psychiatry,” Nat. Hum. Behav. 5(4), 431–432 (2021). 10.1038/s41562-021-01085-w 33820977

[r175] Cearns M. Hahn T. Baune B. T., “Recommendations and future directions for supervised machine learning in psychiatry,” Transl. Psychiatry 9(1), 271 (2019). 10.1038/s41398-019-0607-2 31641106 PMC6805872

[176] Norgeot B. et al., “Minimum information about clinical artificial intelligence modeling: the MI-CLAIM checklist,” Nat. Med. 26(9), 1320–1324 (2020).1078-8956 10.1038/s41591-020-1041-y 32908275 PMC7538196

[177] Ioannidis J. P. A., “Why most published research findings are false,” PLoS Med. 2(8), e124 (2005).1549-1676 10.1371/journal.pmed.0020124 16060722 PMC1182327

[178] Szollosi A. Donkin C., “Arrested theory development: the misguided distinction between exploratory and confirmatory research,” Perspect. Psychol. Sci. 16(4), 717–724 (2021). 10.1177/1745691620966796 33593151

[179] Devezer B. et al., “The case for formal methodology in scientific reform,” R. Soc. Open Sci. 8(3), 200805 (2021). 10.1098/rsos.200805 34035933 PMC8101540

[180] Steegen S. et al., “Increasing transparency through a multiverse analysis,” Perspect. Psychol. Sci. 11(5), 702–712 (2016). 10.1177/1745691616658637 27694465

[181] Piwowar H. A. Vision T. J., “Data reuse and the open data citation advantage,” PeerJ 1, e175 (2013). 10.7717/peerj.175 24109559 PMC3792178

[r182] Tenopir C. et al., “Data sharing by scientists: practices and perceptions,” PLoS One 6(6), e21101 (2011). POLNCL 1932-6203 10.1371/journal.pone.0021101 21738610 PMC3126798

[183] Molloy J. C., “the open knowledge foundation: open data means better science,” PLoS Biol. 9(12), e1001195 (2011). 10.1371/journal.pbio.1001195 22162946 PMC3232214

[184] Bannier E. et al., “The open brain consent: informing research participants and obtaining consent to share brain imaging data,” Hum. Brain Mapp. 42(7), 1945–1951 (2021). HBRME7 1065-9471 10.1002/hbm.25351 33522661 PMC8046140

[185] Wilkinson M. D. et al., “The FAIR guiding principles for scientific data management and stewardship,” Sci. Data 3, 160018 (2016). 10.1038/sdata.2016.18 26978244 PMC4792175

[186] Tucker S. et al., “Introduction to the shared near infrared spectroscopy format,” Neurophotonics 10(01), 013507 (2022). 10.1117/1.NPh.10.1.013507 36507152 PMC9732807

[187] Clark D. Gorgolewski K. J. Cameron Craddock R., “Integrating the brain imaging data structure (BIDS) standard into C-PAC,” GigaScience 5(suppl_1), 3–4 (2016). 10.1186/s13742-016-0147-0-c26823972

[188] Luke R. et al., fNIRS-BIDS, the Brain Imaging Data Structure Extended to Functional Near-Infrared Spectroscopy. Preprint on Open Science Framework, https://osf.io/7nmcp/ (2023).

[189] Foster E. D. Deardorff A., “Open science framework (OSF),” J. Med. Libr. Association 105(2), 203–206 (2017). 10.5195/jmla.2017.88

[190] https://bids.neuroimaging.io/.

[191] http://bids.neuroimaging.io/bep030.

[192] https://openfnirs.org/data/.

[193] https://openneuro.org/.

[194] https://www.nitrc.org/xnat/index.

[195] https://brainlife.io/.

[196] https://about.gitlab.com.

[197] https://bitbucket.org/.

[198] https://osf.io/.

[199] https://www.incf.org/resources/sbps.

[200] https://fairsharing.org/.

[201] Eckersley P. et al., “Neuroscience data and tool sharing: a legal and policy framework for neuroinformatics,” Neuroinformatics 1(2), 149–165 (2003).1539-2791 10.1007/s12021-003-0002-1 15046238

[202] Vlahou A. et al., “Data sharing under the General Data Protection Regulation: time to harmonize law and research ethics?” Hypertension 77(4), 1029–1035 (2021). 10.1161/HYPERTENSIONAHA.120.16340 33583200 PMC7968961

[203] Care Quality Commission, “Anonymisation guidance V1.0,” Anonymising data: key questions for consideration, https://www.cqc.org.uk/sites/default/files/Anonymisation%20Guidance.pdf (accessed 27 May 2022).

[204] Spengler H. Prasser F., “Protecting biomedical data against attribute disclosure,” Stud. Health Technol. Inform. 267, 207–214 (2019). SHTIEW 0926-9630 10.3233/SHTI190829 31483274

[205] Haber A. C. et al., “Open tools for quantitative anonymization of tabular phenotype data: literature review,” Brief Bioinform. 23(6), bbac440 (2022). 10.1093/bib/bbac440 36215114 PMC9677485

